# Purification and *In Vitro* Activity of Mitochondria Targeted Nitrogenase Cofactor Maturase NifB

**DOI:** 10.3389/fpls.2017.01567

**Published:** 2017-09-12

**Authors:** Stefan Burén, Xi Jiang, Gema López-Torrejón, Carlos Echavarri-Erasun, Luis M. Rubio

**Affiliations:** Centro de Biotecnología y Genómica de Plantas (CBGP), Universidad Politécnica de Madrid (UPM), Instituto Nacional de Investigación y Tecnología Agraria y Alimentaria (INIA) Madrid, Spain

**Keywords:** nitrogen fixing plants, yeast, mitochondria, SAM-radical, iron-molybdenum cofactor

## Abstract

Active NifB is a milestone in the process of engineering nitrogen fixing plants. NifB is an extremely O_2_-sensitive *S*-adenosyl methionine (SAM)–radical enzyme that provides the key metal cluster intermediate (NifB-co) for the biosyntheses of the active-site cofactors of all three types of nitrogenases. NifB and NifB-co are unique to diazotrophic organisms. In this work, we have expressed synthetic codon-optimized versions of NifB from the γ-proteobacterium *Azotobacter vinelandii* and the thermophilic methanogen *Methanocaldococcus infernus* in *Saccharomyces cerevisiae* and in *Nicotiana benthamiana*. NifB proteins were targeted to the mitochondria, where O_2_ consumption is high and bacterial-like [Fe-S] cluster assembly operates. In yeast, NifB proteins were co-expressed with NifU, NifS, and FdxN proteins that are involved in NifB [Fe–S] cluster assembly and activity. The synthetic version of thermophilic NifB accumulated in soluble form within the yeast cell, while the *A. vinelandii* version appeared to form aggregates. Similarly, NifB from *M. infernus* was expressed at higher levels in leaves of *Nicotiana benthamiana* and accumulated as a soluble protein while *A. vinelandii* NifB was mainly associated with the non-soluble cell fraction. Soluble *M. infernus* NifB was purified from aerobically grown yeast and biochemically characterized. The purified protein was functional in the *in vitro* FeMo-co synthesis assay. This work presents the first active NifB protein purified from a eukaryotic cell, and highlights the importance of screening *nif* genes from different organisms in order to sort the best candidates to assemble a functional plant nitrogenase.

## Introduction

Agricultural systems in developed countries are largely based on cereal crops, which provide most of the calories and proteins in the human diet (Borlaug, 2002). Nitrogen and water availability are the most important factors limiting cereal crop productivity. Over the last 100 years, cereal crop yields have been increased by addition of chemically synthesized nitrogen fertilizers (Smil, 2000; Galloway et al., 2008). However, the extensive use of these commercial nitrogen fertilizers in developed countries poses enormous and pressing environmental threats (Vitousek et al., 1997). On the other hand, the cost of chemical fertilizers is prohibitive for poor farmers, and they are scarcely used in most of Africa with consequence of poverty and hunger derived from extremely low crop yields (Sanchez and Swaminathan, 2005). During the last years, the scientific community has paid considerable attention to this problem and acknowledged the need of disruptive technological changes. One way to tackle this problem could be the generation of so-called nitrogen-fixing plants, aimed to solve the nitrogen problem (Stokstad, 2016; Vicente and Dean, 2017).

Nitrogen fixation is the conversion of inert atmospheric N_2_ into NH_3_, a biologically active form of nitrogen. Only a few bacteria are naturally able to fix nitrogen through the activity of an enzyme called nitrogenase; collectively known as diazotrophs (nitrogen eaters). In this regard, transferring bacterial nitrogen fixation (*nif*) genes into the plant genome could result in plants able to fix nitrogen, and therefore in crops less dependent of external nitrogen fertilization. This direct approach eliminates the need to generate or optimize interactions of cereals with specific symbiotic or associative nitrogen fixing bacteria (Oldroyd and Dixon, 2014). However, two main barriers are believed to impair the direct *nif* gene transfer approach (Curatti and Rubio, 2014): the known sensitivity of nitrogenase to O_2_ and the genetic complexity of nitrogenase biosynthesis. Two decades of genetic and biochemical analyses culminated with the unambiguous identification of the essential proteins required for nitrogenase cofactor biosynthesis (Curatti et al., 2007). On the contrary, overcoming the O_2_ sensitivity barrier in plants remains largely unexplored.

Nitrogenases have two O_2_-sensitive protein components: a dinitrogenase that catalyzes the nitrogen fixation reaction and a dinitrogenase reductase that serves as obligate electron donor to dinitrogenase (Bulen and LeComte, 1966). In the case of the widespread molybdenum nitrogenase these components are called Fe protein and MoFe protein. The Fe protein is a homodimer of the *nifH* gene product that contains a [4Fe–4S] cluster at the subunit interface. The MoFe protein is a heterotetramer of the *nifD* and *nifK* gene products that contains two complex iron–sulfur (Fe–S) clusters called iron-molybdenum cofactor (FeMo-co) and P-cluster. The type of [4Fe–4S] cluster found in NifH is ubiquitous in nature. In fact, plants carry [Fe–S] cluster assembly machineries in mitochondria, chloroplasts, and cytosol, which are all capable of synthesizing [4Fe–4S] clusters (Balk and Pilon, 2011). However, the P-cluster and FeMo-co are unique to diazotrophs. Their uniqueness implies that specialized cellular biosynthetic pathways, involving multiple *nif* gene products, are required for cofactor synthesis and NifDK maturation (Rubio and Ludden, 2008).

Successful expression and maturation of the prokaryotic nitrogenase protein in a eukaryotic host, in order to develop N_2_ fixing cereal crops, could revolutionize agricultural systems worldwide. For this to succeed, a deeper understanding of the processes involved in the formation of active nitrogenase in a eukaryotic cell is required. In this regard, expression of *nif* genes in *Saccharomyces cerevisiae* have shown that: (1) active NifH can be achieved upon mitochondrial targeting (Lopez-Torrejon et al., 2016), providing a proof of concept that O_2_-sensitive Nif proteins can be assembled in an eukaryotic cell organelle, and (2) that expression and mitochondria targeting of nine Nif proteins (NifUSHMDKBEN) resulted in proper mitochondria targeting, processing and NifDK tetramer formation (Burén et al., 2017), an essential step of nitrogenase assembly. However, to obtain similar results in a plant cell background is likely to be more challenging, as the O_2_ generated during photosynthesis could create even a harsher environment for nitrogenase proteins, especially in the chloroplast. This was recently suggested from work by Ivleva et al. (2016), where Fe protein activity from transplastomic *Nicotiana tabacum* plants only could be detected at very low levels in plants previously incubated at sub-ambient O_2_ (Ivleva et al., 2016). Importantly, a recent study showed that 16 mitochondria targeted Nif proteins from *Klebsiella pneumoniae* (among them NifB) could be successfully expressed in leaves of *Nicotiana benthamiana* (Allen et al., 2017). Although no protein activities were reported, this work showed that most Nif proteins were well expressed and accumulated at their estimated sizes within the plant tissue, with the exception of NifD that appeared to be processed to a polypeptide of smaller size, as has also been observed in yeast (Burén et al., 2017).

A main hurdle to overcome in order to generate functional nitrogenase proteins is obtaining active NifB. NifB is an extremely O_2_-sensitive *S*-adenosyl methionine (SAM)–radical enzyme (Curatti et al., 2006), that provides the key intermediate metal cluster (called NifB-co) in the biosynthesis of FeMo-co (Shah et al., 1994; Guo et al., 2016). As NifB-co also serves as precursor for FeV-co in the vanadium nitrogenase and for FeFe-co in the iron-only nitrogenase, NifB is required for all biological nitrogen fixation activity in nature (Bishop and Joerger, 1990; Curatti et al., 2007; Dos Santos et al., 2012). Because of its O_2_ sensitivity and instability, and since it is unlikely that NifB or NifB-co can be replaced by components of plant origin, plant cell NifB-co accumulation is likely to be one of the main barriers in the generation of an active plant nitrogenase (Curatti and Rubio, 2014; Vicente and Dean, 2017).

In this work, two naturally occurring NifB proteins, from the model-diazotroph *Azotobacter vinelandii* and from the thermophilic methanogen *Methanocaldococcus infernus* (see accompanying paper by Arragain et al., submitted manuscript), were expressed in *S. cerevisiae* and targeted to mitochondria. Mitochondria was chosen due to the high rate of O_2_ consumption, and the plentiful ATP and reducing power generated by respiration (Curatti and Rubio, 2014), in addition to the bacterial-like [Fe–S] cluster assembly machinery available (Lill and Muhlenhoff, 2008). NifB proteins were co-expressed with NifU, NifS, and FdxN proteins, involved in NifB [Fe–S] cluster formation and activity (Yuvaniyama et al., 2000; Johnson et al., 2005; Zhao et al., 2007; Jiménez-Vicente et al., 2014). Surprisingly, only NifB from the thermophile was found to accumulate in a soluble form, while NifB from *A. vinelandii* appeared to form aggregates. The soluble *M. infernus* NifB was purified and proven functional in the *in vitro* FeMo-co synthesis assay. *A. vinelandii* and *M. infernus* NifB were also targeted to the mitochondria in leaf cells of *N. benthamiana* (tobacco). As in yeast, the synthetic version of NifB from *M. infernus* was better expressed and accumulated as a soluble protein while the *A. vinelandii* NifB was mainly associated with the non-soluble cell fraction. These results underline the importance of screening for functionality each one of the Nif proteins required to mature nitrogenase.

## Results

### Generation of Yeast Platform Strains for NifB Expression

Synthetic versions of *A. vinelandii nifB (nifB_Av_)* and *M. infernus nifB* (*nifB_Mi_*) were codon-optimized for *S. cerevisiae* and cloned into expression vectors under the control of the galactose inducible promoters (Supplementary Figures S1, S2). As NifU, NifS, and FdxN participate in NifB maturation and activity (Zhao et al., 2007; Jiménez-Vicente et al., 2014), synthetic versions of the *A. vinelandii nifU*, *nifS*, and *fdxN* genes, codon-optimized for *S. cerevisiae*, were additionally cloned into expression vectors under the control of the galactose inducible promoters. To ensure mitochondria targeting of the expressed proteins, SU9 leader sequences, previously shown to deliver yeast expressed Nif proteins to the mitochondria (Burén et al., 2017) were added in-frame and upstream of each gene. To facilitate NifB purification by affinity chromatography, the coding sequence for 10 histidines were added 3′ of each *nifB* gene. Expression vectors were co-introduced into *S. cerevisiae* strain W303-1a, generating yeast strains SB09Y and SB10Y for the expression of mitochondria targeted NifU, NifS, and FdxN, together with NifB*_Av_*-His_10_ or NifB*_Mi_*-His_10_ (hereafter called yNifB*_Av_* and yNifB*_Mi_*), respectively (**Table 1**).

**Table 1:** Yeast expression plasmids and yeast strains used in this work.

Strain	Transformed plasmids	Expressed proteins	Full-length (kDa)	Processed (kDa)
SB09Y	pN2GLT4	SU9-NifU	40.6	33.6
		SU9-NifS	51.0	44.0
	pN2SB22	SU9-FdxN	17.1	9.8
		SU9-NifB*_Av_*-His_10_	63.6	56.3
SB10Y	pN2GLT4	SU9-NifU	40.6	33.6
		SU9-NifS	51.0	44.0
	pN2SB24	SU9-FdxN	17.1	9.8
		SU9-NifB*_Mi_*-His_10_	43.8	36.4
SB03Y	pN2SB15	SU9-FdxN	17.1	9.8
		SU9-His_10_-NifB*_Av_*	63.6	56.3
SB12Y	pN2GLT4	SU9-NifU	40.6	33.6
		SU9-NifS	51.0	44.0
	pN2SB39	SU9-FdxN-HA	18.2	10.9
		SU9-NifB*_Mi_*-His_10_	43.8	36.4


*Nitrogenase-related proteins and their expected sizes when expressed in yeast from expression vectors generated in this study. Processed forms refer to sizes following predicted SU9 cleavage (RAY-SS, (Vögtle et al., 2009)). See Supplementary Figures S1, S2 for details.*

### NifB Expression, Mitochondria Targeting, and Solubility

**FIGURE 1:**
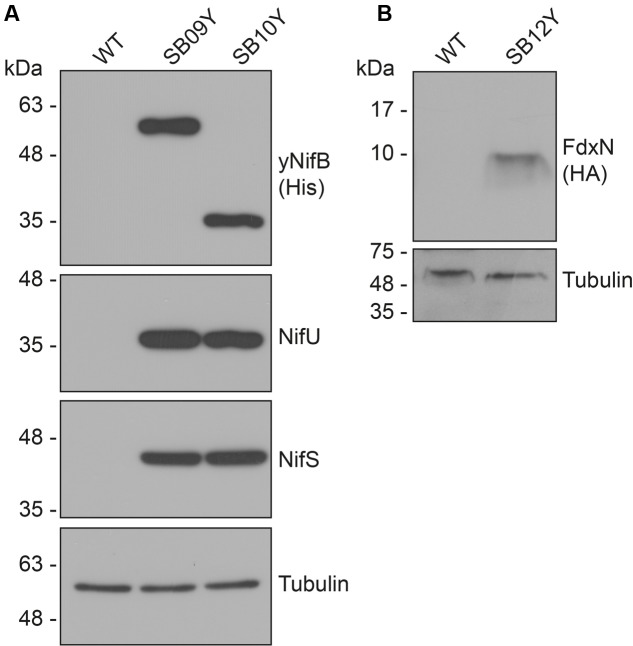
Expression of NifB, NifU, NifS and FdxN proteins in *S. cerevisiae*. **(A)** Western blot analysis of yNifB*_Av_* and yNifB*_Mi_*, as well as NifU and NifS, in total protein extracts from strains SB09Y and SB10Y. **(B)** Western blot analysis of C-terminally HA-tagged FdxN in total protein extract from SB12Y strain. Extracts in (A) and (B) were prepared from aerobically grown cells following galactose induction, and proteins in the extract separated by SDS-PAGE before transferred to membranes. Antibodies recognizing NifU*_Av_*, NifS*_Av_*, His epitope, HA epitope, and tubulin were used. Tubulin immunoblot signal intensity is used as loading control. See **Table 1** for details about recombinant yeast strains.

Western blot analysis of yeast cell-free extracts using antibodies specifically recognizing NifU, NifS, and histidine-tag (for yNifB*_Av_* or yNifB*_Mi_*) confirmed expression of all these proteins in SB09Y and SB10Y strains grown aerobically with galactose as inducer. Protein migrations in SDS-PAGE were consistent with efficient mitochondria leader sequence processing (**Table 1** and **Figure 1A**). Detection of FdxN in SB09Y and SB10Y was difficult, presumably due to the small size of FdxN (10 kDa) and/or to weak binding of FdxN antibodies generated for this study. To confirm that FdxN was successfully expressed from the GAL10 promoter, presence of *fdxN* transcripts were verified in SB09Y and SB10Y strains (Supplementary Figure S3). In addition, an epitope-tagged version of the protein where a C-terminal HA-tag was added to the SU9-FdxN construct was generated and expressed in strain SB12Y (**Figure 1B**).

**FIGURE 2:**
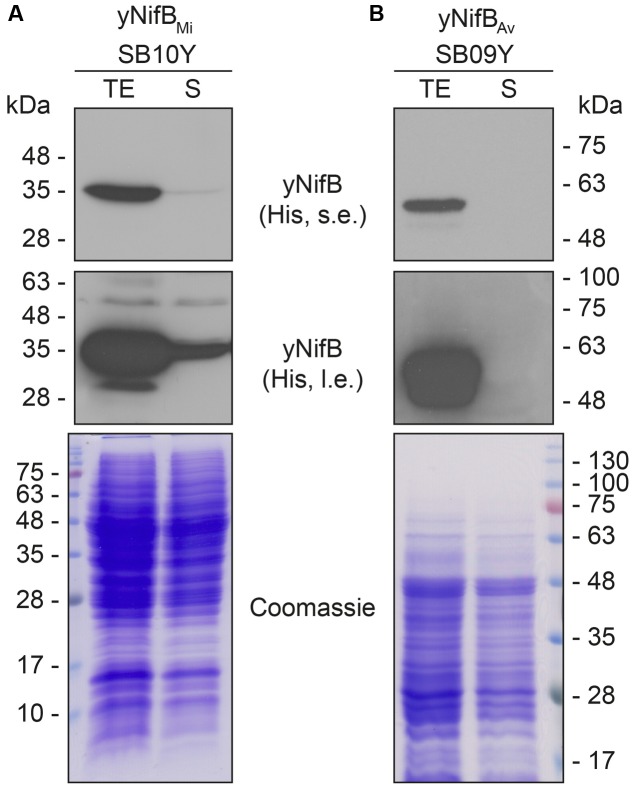
Solubility of *A. vinelandii* and *M. infernus* NifB proteins expressed in *S. cerevisiae*. **(A,B)** Western blot analysis of yNifB*_Mi_* (A) and yNifB*_Av_* (B) present in total protein extracts (TE) and the soluble fraction (S) of yeast strains SB10Y and SB09Y. Conditions for strain growth, induction of protein expression, total extract preparation, and separation by SDS-PAGE are as in **Figure 1**. Antibodies recognizing the His epitope were used. Short (s.e.) and long (l.e.) film exposures are shown. Coomassie stained SDS gels (below) of the protein extracts are included as loading controls.

**FIGURE 3:**
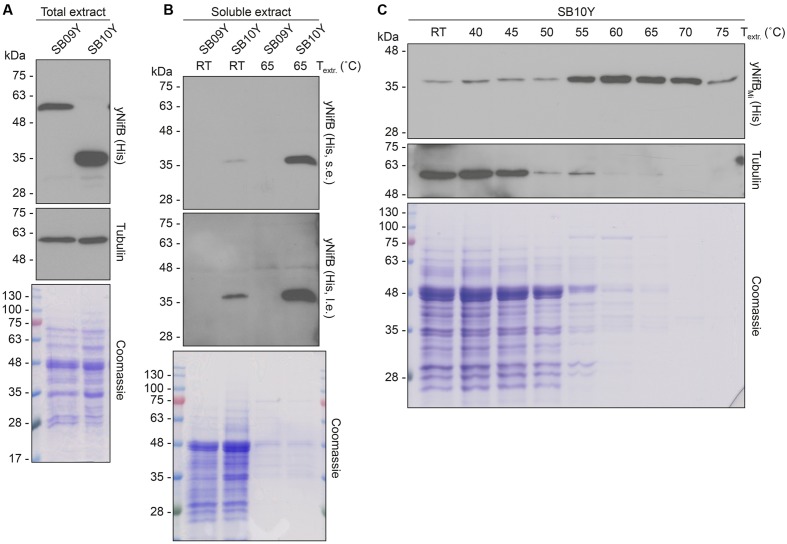
Levels of soluble yNifB*_Av_* and yNifB*_Mi_* in heat-treated yeast extracts. **(A,B)** Total NifB levels (A) and levels of soluble NifB upon 65°C heat-treatment (B) of protein extracts from yeast expressing yNifB*_Av_* (SB09Y) or yNifB*_Mi_* (SB10Y). Antibodies recognizing the His epitope were used. Short (s.e.) and long (l.e.) film exposures are shown. RT means room temperature. Tubulin and/or Coomassie stained SDS gels of the same protein extracts are included as loading controls. **(C)** Western blot analysis of soluble yNifB*_Mi_* in SB10Y protein extracts upon heat-treatment at increasing temperatures. Heat-induced precipitation of yeast proteins in the extract at the different temperatures is shown using antibodies recognizing tubulin, as well as by Coomassie staining of proteins from the extract resolved by SDS-PAGE.

Further analysis of the soluble fractions prepared from yeast cells lysed in absence of detergents indicated that most yNifB*_Mi_* and nearly all yNifB*_Av_* were of poor solubility (**Figure 2**). This suggested that the proteins were either forming insoluble aggregates upon strong expression or interacting with membranes. A similar behavior was observed for NifB*_Av_* and NifB*_Ko_* (*Klebsiella oxytoca* NifB) overexpressed in *Escherichia coli* (data not shown). Exchanging the C-terminal His-tag for an N-terminal variant, and addition of detergents during lysis (see Materials and Methods for details), did not improve solubility (Supplementary Figure S4). As NifB from the thermophile *M. infernus* previously showed heat-resistant properties (Wilcoxen et al., 2016), several distinct extraction conditions were tested (including different temperatures) to screen yNifB*_Mi_* solubility and to find a protocol for extraction and enrichment of yNifB*_Mi_*. While increased concentration of glycerol did not improve solubility, the pH of the extraction buffer was important (Supplementary Figure S5). In addition, exposing the total yeast lysate to elevated temperatures before centrifugation not only reduced the amount of total yeast proteins remaining in solution, but also increased the levels of yNifB*_Mi_* in the soluble fraction of the extract. Unfortunately, no similar improvement could be obtained for yNifB*_Av_* (**Figures 3A,B** and Supplementary Figure S6) impairing yNifB*_Av_* purifications. Further optimization confirmed that maximum solubility yNifB*_Mi_* was obtained at pH 8 upon treatment at 60–65°C (**Figure 3C**). Therefore 65°C was chosen for the following yNifB*_Mi_* extractions in order to minimize the complexity of the yeast cell-free extracts used for affinity chromatography.

### Yeast-Expressed NifB*_Mi_* Is Active in the *In Vitro* FeMo-co Synthesis Assay

**FIGURE 4:**
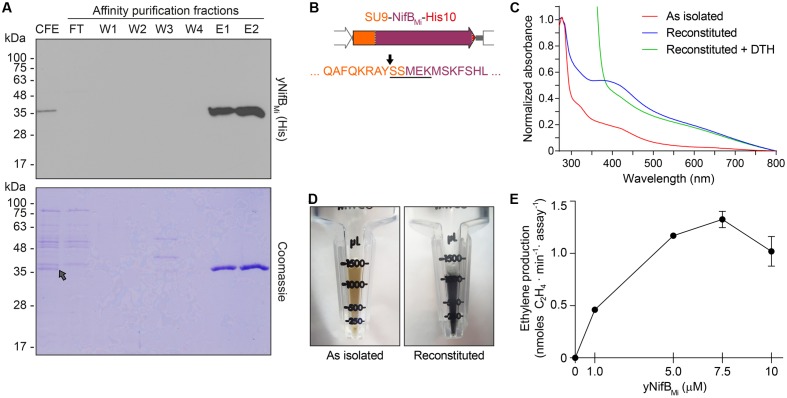
Purification and biochemical properties of yNifB*_Mi_*. **(A)** SDS-PAGE and Western blot analysis of yNifB*_Mi_* purification. CFE, 65°C heated SB10Y cell-free extract; FT, affinity chromatography flow through; W1-W4 and E1-E2, affinity chromatography wash and elution fractions containing increasing concentrations of imidazole (see Materials and Methods for details). Grey arrow in the Coomassie stained panel points to the position of yNifB*_Mi_* in the gel. **(B)** SU9 processing site (black arrow) of yNifB*_Mi_*. Underlined sequence indicates the N-terminal amino acids of yNifB*_Mi_* identified by Edman degradation. **(C)** UV-visible spectra of as isolated, reconstituted, and dithionite (DTH)-reduced reconstituted yNifB*_Mi_*. **(D)** Typical color of as isolated and reconstituted yNifB*_Mi_* purified preparations. **(E)** Titration of FeMo-co synthesis and nitrogenase reconstitution assay with yNifB*_Mi_*. The indicated concentrations of yNifB*_Mi_* monomer were used. NifB activity was determined by acetylene reduction assay of reconstituted NifDK from *ΔnifB A. vinelandii* UW140 cell-free extracts. Data represent mean ± standard deviation (*n* = 2) at each yNifB*_Mi_* concentration.

Typical yeast NifB*_Mi_* purification yielded about 4 mg/100 g cell pellet (4.4 ± 1.1, mean and standard deviation from four individual purifications), and yNifB*_Mi_* was at near purity as determined by SDS-PAGE analysis (**Figure 4A**). To confirm mitochondria import and functionality of the SU9 leader sequence, purified yNifB*_Mi_* was subjected to N-terminal sequencing. Successful processing of the SU9 sequence was verified, and cleavage appeared at the site predicted from alignment of the SU9 peptide with a reported consensus sequence for yeast mitochondria proteins (Vögtle et al., 2009) (**Figure 4B**). While as isolated yNifB*_Mi_* showed some color and UV-vis absorbance spectrum characteristic of Fe–S protein (3.3 ± 0.8 Fe atoms per monomer from four individual purifications, S not determined), *in vitro* reconstitution with Fe and S increased color intensity and the 320 and 420 nm features of the UV-vis spectrum indicative of [4Fe–4S] cluster formation (**Figures 4C,D**). Treatment with dithionite (DTH) reduced absorbance at 420 nm as expected for a redox responsive Fe–S protein. Fe and S content of reconstituted yNifB*_Mi_* was consistent with the presence of, at minimum, two [Fe-S] clusters in addition to the SAM-binding [4Fe-4S] cluster (12.5 ± 2.8 Fe and 10.6 ± 3.1 S atoms per monomer; average ± standard deviation from four individual purifications). All these features are typical of NifB proteins (Curatti et al., 2006; Wilcoxen et al., 2016).

NifB can be used for *in vitro* FeMo-co synthesis and nitrogenase reconstitution assays using cell-free extracts of Δ*nifB A. vinelandii*, supplemented with ATP-regenerating mixture, molybdenum (Mo), and homocitrate (Curatti et al., 2006). When *in vitro* FeMo-co synthesis occurs, *de novo*-synthesized FeMo-co is incorporated into apo-MoFe nitrogenase present in the extract and activity of reconstituted nitrogenase can be determined by the acetylene reduction assay. To test whether reconstituted yNifB*_Mi_* was functional, 5 μM protein was added to UW140 extracts lacking NifB-co activity, but providing the rest of the protein components required for FeMo-co synthesis and activatable apo-MoFe nitrogenase. While extract without yNifB*_Mi_* only showed negligible acetylene reduction, addition of yNifB*_Mi_* resulted in 40-fold increase in ethylene formation (**Table 2**). Importantly, yNifB*_Mi_* showed similar concentration-dependent activity as purified and reconstituted NifB from *A. vinelandii* (Curatti et al., 2006) (**Figure 4E**). The maximum activity appeared to occur at slightly higher concentration (5 μM vs. 1 μM), which could result from slight incompatibility between the yNifB*_Mi_* and the other Nif components in the UW140 *A. vinelandii* extract, as has been shown for NifH (Emerich and Burris, 1976, 1978), or from the suboptimal reaction temperature for the thermophile *M. infernus* (optimal growth at 85°C) NifB protein (Jeanthon et al., 1998).

**Table 2:** yNifB*_Mi_*-dependent *in vitro* FeMo-co synthesis and nitrogenase reconstitution assays.

UW140	nmol C_2_H_4_ (min^-1^ ⋅ assay^-1^)
- NifB-co	0.04
+ NifB-co	15.54 ± 0.23
+ yNifB*_Mi_* (1)	1.33 ± 0.22
+ yNifB*_Mi_* (2)	0.97 ± 0.02
+ yNifB*_Mi_* (3)	1.92 ± 0.04
+ yNifB*_Mi_* (4)	1.41 ± 0.06


*Acetylene reduction assays of nitrogenase reconstituted in Δ*nifB* A. vinelandii extracts. UW140 extracts, or UW140 extracts supplemented with NifB-co, were used as negative and positive controls, respectively. Data represent mean ± standard deviation (n = 2) from four individual yNifB_*Mi*_ purifications (at 5 μM yNifB_*Mi*_).*

In summary, yNifB*_Mi_* exhibits the spectroscopic and catalytic properties of active NifB proteins. Further studies will aim to determine whether the yNifB*_Mi_* protein can support NifB-co synthesis *in vivo* in mitochondria of yeast.

### Expression and Mitochondria Targeting of NifB in Plant Leaves

In a work by Allen et al. (2017), 16 HA-tagged nitrogenase proteins from *K. oxytoca* were separately expressed in *Agrobacterium tumefaciens* infiltrated *N. benthamiana* leaves, and targeted to the mitochondria. *K. oxytoca* NifB was one of the Nif proteins that resulted in highest protein expression level (Allen et al., 2017). However, as leaf proteins were extracted in SDS buffer upon heating, the level of soluble NifB protein is difficult to estimate. In order to test whether differences in solubility of plant expressed and mitochondria targeted NifB proteins could be observed, as in yeast, NifB*_Av_* and NifB*_Mi_* were cloned into plant expression vectors under the control of the constitutive 35S promoter (Supplementary Figures S7, S8). As yeast and tobacco codon usage is similar, no further sequence optimization and gene synthesis was performed.

**FIGURE 5:**
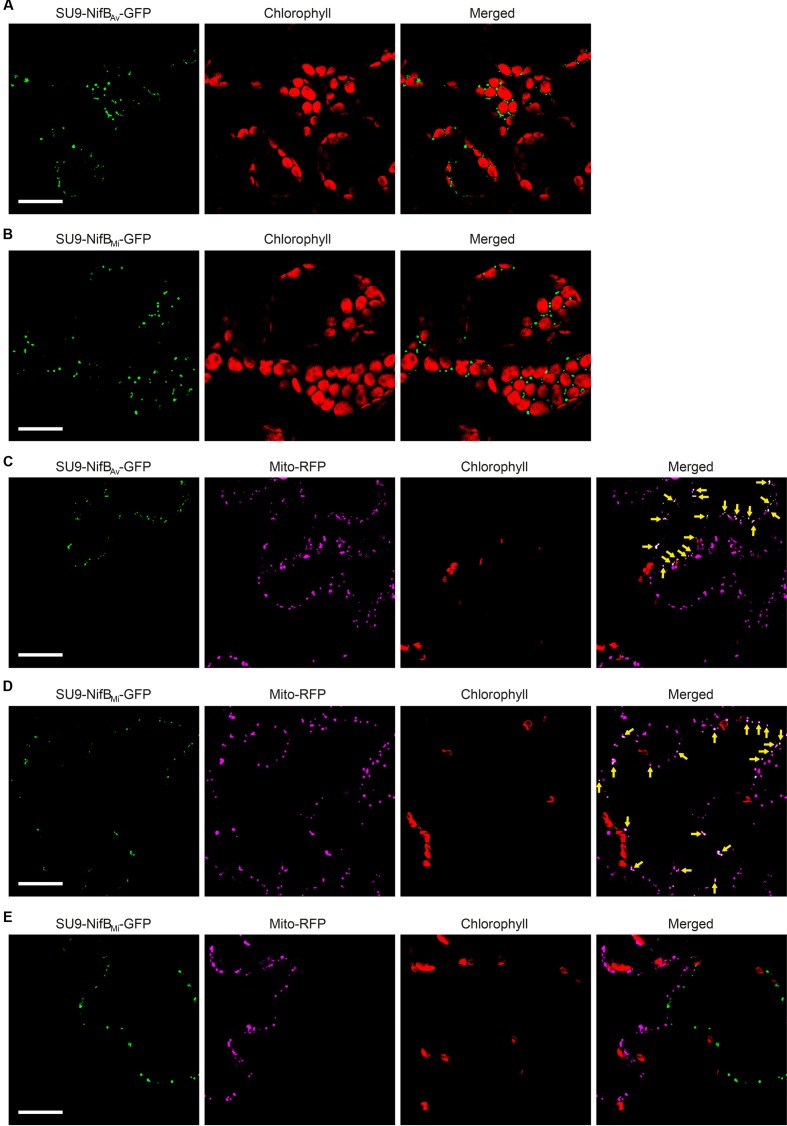
Expression of mitochondria targeted (SU9) NifB*_Av_* and NifB*_Mi_* GFP fusions in *N. benthamiana* leaves. **(A,B)** Mesophyll cells expressing SU9-NifB*_Av_*-GFP (A) or SU9-NifB*_Mi_*-GFP (B). GFP (green) and chlorophyll autofluorescence (red) of chloroplasts is shown. **(C–E)** Epidermal cells expressing SU9-NifB*_Av_*-GFP (C) and SU9-NifB*_Mi_*-GFP (D,E), together with a fluorescent mitochondria marker (Mito-RFP). GFP (green), Mito-RFP (magenta) and chlorophyll autofluorescence (red) of chloroplasts is shown. Co-localization of SU9-NifB*_Av_*-GFP or SU9-NifB*_Mi_*-GFP constructs with Mito-RFP labeled structures is shown as white in the merged images, and highlighted with yellow arrows. Adjacent cells expressing SU9-NifB*_Mi_*-GFP or Mito-RFP are shown as control to verify the specificity of the signal recorded in each channel (E). Scale bars show 30 μm. Confocal Microscopy conditions are specified in Materials and Methods.

**FIGURE 6:**
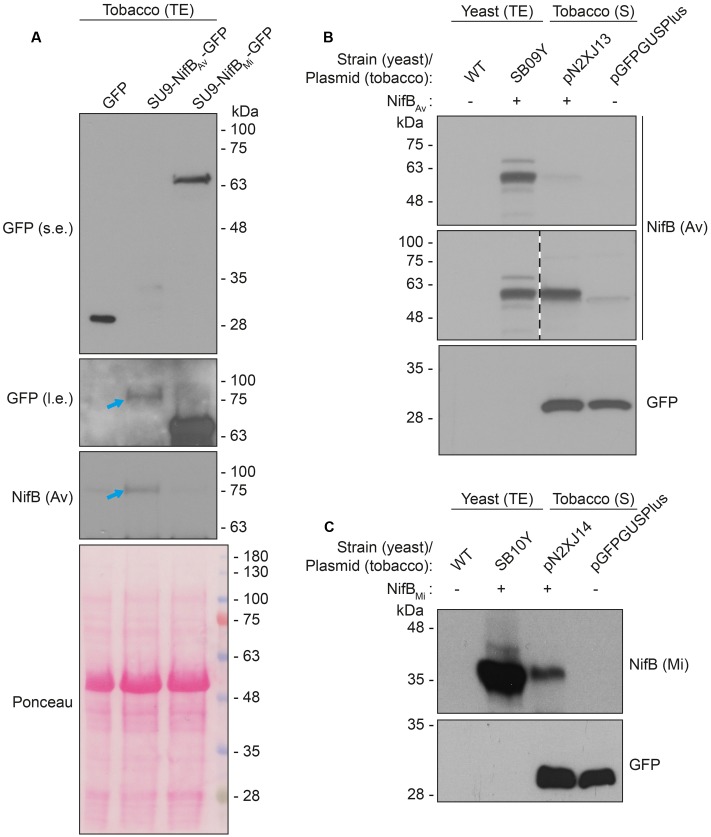
Expression and solubility of mitochondria targeted (SU9) NifB*_Av_* and NifB*_Mi_* in *N. benthamiana* leaves. **(A)** Western blot analysis of total protein extracts (TE) prepared from infiltrated *N. benthamiana* leaves expressing GFP, SU9-NifB*_Av_*-GFP or SU9-NifB*_Mi_*-GFP. Blue arrows indicate the polypeptide recognized both by GFP and NifB*_Av_* specific antibodies. Short (s.e.) and long (l.e.) film exposures of the GFP antibody probed membrane are shown. **(B)** Migration of SU9-NifB*_Av_*-His_10_ when expressed in *S. cerevisiae* and *N. benthamiana*. Migration in SDS-PAGE was determined after Western blot analysis using NifB*_Av_* specific antibodies. Total protein extracts (TE) from W303-1a *S. cerevisiae* cells (WT) or cells expressing SU9-NifB*_Av_*-His_10_ (SB09Y) were prepared. Soluble protein extracts (S) from *N. benthamiana* leaf cells infiltrated with *A. tumefaciens* containing control vector (pGFPGUSPlus) or vector for expression of SU9-NifB*_Av_*-His_10_ (pN2XJ13). Dotted line indicate different exposures of the right part of the membrane. See Supplementary Figure S9 for entire gel of the cropped exposure. **(C)** Migration of SU9-NifB*_Mi_*-His_10_ when expressed in *S. cerevisiae* and *N. benthamiana*. Migration in SDS-PAGE was determined after Western blot analysis using NifB*_Mi_* specific antibodies. Total protein extracts (TE) from W303-1a *S. cerevisiae* cells (WT) or cells expressing SU9-NifB*_Mi_*-His_10_ (SB10Y) were prepared. Soluble protein extracts (S) from *N. benthamiana* leaf cells infiltrated with *A. tumefaciens* containing control vector (pGFPGUSPlus) or vector for expression of SU9-NifB*_Mi_*-His_10_ (pN2XJ14). As control of *N. benthamiana* leaf infiltration, GFP expressed from the pGFPGUSPlus vector backbone was detected (B,C).

As SU9 is a mitochondria leader sequence from fungi without obvious plant homolog, the C-terminal His-tag of SU9-NifB*_Av_* and SU9-NifB*_Mi_* was replaced with GFP to track SU9 functionality in *N. benthamiana* cells. Confocal microscopy analysis showed that SU9 successfully targeted the two NifB variants to the mitochondria of *N. benthamiana*, as seen from co-localization with a red fluorescent mitochondria marker (Candat et al., 2014) (**Figures 5A–D**). Specific and individual detection of the fluorescent signals was verified from adjacent cells expressing only each one of the constructs (**Figure 5E**). Confocal microscopy indicated that the expression level of SU9-NifB*_Av_*-GFP was lower than SU9-NifB*_Mi_*-GFP, which was confirmed by Western blot analysis (**Figure 6A**). Importantly, SU9-NifB*_Mi_*-GFP was only detected in the soluble fraction of the extract, in contrast to SU9-NifB*_Av_*-GFP that could also be seen in the pellet fraction (data not shown). Migration of the expressed fusion proteins was consistent with correct SU9 leader sequence processing in *N. benthamiana* cells (**Figure 6A** and **Table 3**).

**Table 3:** Tobacco expressed nitrogenase related proteins and their expected sizes.

Plasmid	Expressed protein	Full-length (kDa)	Processed (kDa)
pN2XJ13	SU9-NifB*_Av_*-His_10_	64.0	56.7
pN2XJ14	SU9-NifB*_Mi_*-His_10_	44.3	37
pN2XJ15	SU9-NifB*_Av_*-GFP	90.0	82.7
pN2XJ16	SU9-NifB*_Mi_*-GFP	70.3	63
pN2XJ19	COX4-twinStrep-GFP	33.3	30.3
pN2XJ20	COX4-twinStrep- NifB*_Av_*	61.1	58.1
pN2XJ21	COX4-twinStrep- NifB*_Mi_*	41.3	38.3


*Plant expression vectors generated in order to express nitrogenase related proteins. Expressed proteins and their expected sizes are indicated. Processed forms refer to sizes following SU9 (RAY-SSM, **Figure 4**) or COX4 (YLL-QQK, (Vögtle et al., 2009)) cleavage as in yeast. See Supplementary Figures S7, S8, S10 for details.*

Migration of the plant expressed C-terminally His-tagged versions of the SU9-NifB*_Av_* and SU9-NifB*_Mi_* proteins appeared identical to the corresponding proteins expressed in yeast, supporting that the SU9 leader sequence was processed correctly also in the plant mitochondria (**Figures 6B,C** and Supplementary Figure S9).

**FIGURE 7:**
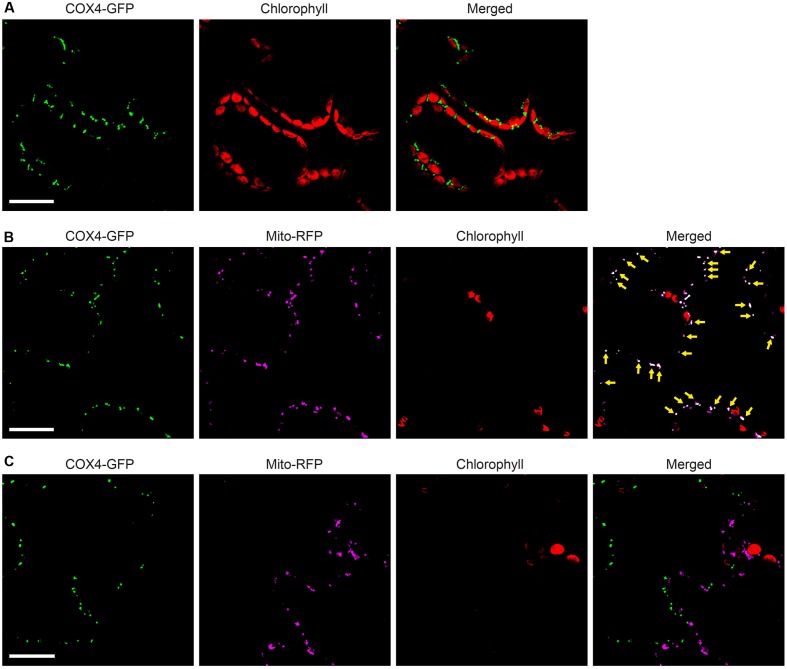
Functionality of COX4 leader sequence for mitochondria targeting of GFP in *N. benthamiana* leaves. **(A)** Mesophyll cells expressing COX4-twinStrep-GFP. GFP (green) and chlorophyll autofluorescence (red) of chloroplasts is shown. **(B,C)** Epidermal cells expressing COX4-twinStrep-GFP together with a fluorescent mitochondria marker (Mito-RFP). GFP (green), Mito-RFP (magenta) and chlorophyll autofluorescence (red) of chloroplasts is shown. Co-localization of COX4-twinStrep-GFP with Mito-RFP labeled structures (B) is shown as white in the merged image, and highlighted with yellow arrows. Adjacent cells expressing COX4-twinStrep-GFP or Mito-RFP (C) are shown as control to verify the specificity of the signal recorded in each channel. Scale bars show 30 μm. Confocal Microscopy conditions are specified in Materials and Methods.

To enable simultaneous and comparative detection of the two *N. benthamiana* expressed NifB variants, and to exclude that solubility was affected by the C-terminal GFP moiety, new constructs were generated were the His-tag was exchanged for an N-terminal 28 amino acid Twin-Strep-tag (Schmidt et al., 2013) (Supplementary Figures S8, S10). The Twin-Strep-tag is an improved version of the eight amino acid Strep-tag II that was shown superior to His-tag for use with plant tissue extracts (Witte et al., 2004). In addition, the SU9 signal was replaced by the first 29 amino acids of the yeast cytochrome c oxidase IV (COX4) protein, which has been shown to successfully target proteins to the mitochondria in tobacco and *Arabidopsis thaliana* (Köhler et al., 1997; Nelson et al., 2007; Pan et al., 2014). As cleavage of COX4 in yeast has been shown to occur between amino acids 25 and 26 (Vögtle et al., 2009), similar processing in *N. benthamiana* would leave only four amino acids in addition to the Twin-Strep-tag. To verify functionality of the COX4 peptide, and to confirm that the Twin-Strep-tag was not interfering with targeting or solubility, a COX4-twin-Strep-GFP construct was generated (Supplementary Figures S8, S10). As expected, COX4 efficiently targeted twin-Strep-GFP to mitochondria in *N. benthamiana* cells (**Figures 7A–C**). Specific and individual detection of the fluorescent signals was verified using adjacent cells expressing only one of the constructs (**Figure 7C**).

**FIGURE 8:**
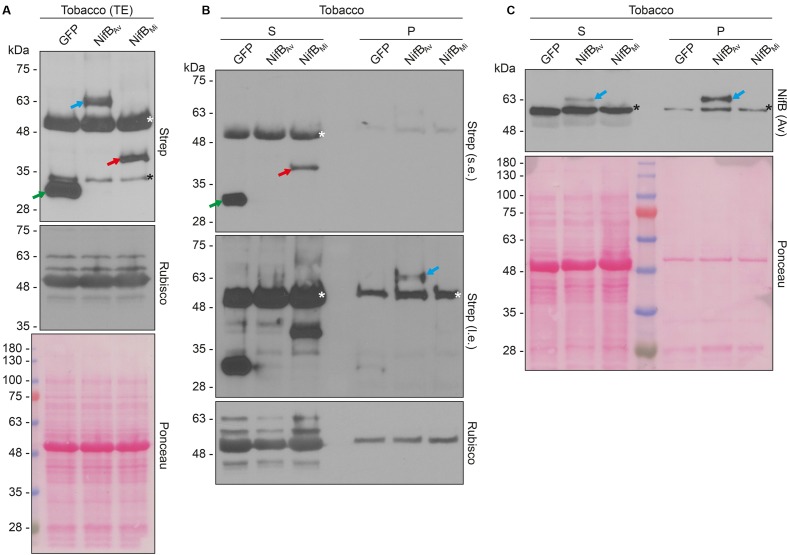
Expression and solubility of mitochondria targeted (COX4) NifB*_Av_* and NifB*_Mi_* in *N. benthamiana* leaves. **(A)** Western blot analysis of total protein extracts (TE) prepared from infiltrated *N. benthamiana* leaves expressing COX4-twinStrep-GFP (GFP), COX4-twinStrep-NifB*_Av_* (NifB*_Av_*) or COX4-twinStrep-NifB*_Mi_* (NifB*_Mi_*) and separated by SDS-PAGE. The COX4-twinStrep-GFP (green arrow), COX4-twinStrep-NifB*_Av_* (blue arrow), COX4-twinStrep-NifB*_Mi_* (red arrow) proteins are highlighted. A pronounced non-specific polypeptide detected using the Strep-tag antibodies (white star) co-migrated with the large subunit of Rubisco. The membrane probed with antibodies against Rubisco was also stained with Ponceau and is included as loading control. **(B,C)** Western blot analysis of the soluble (S) and non-soluble pellet (P) fractions of *N. benthamiana* leaf total extracts used in (A), using Strep-tag antibodies (B) or NifB*_Av_* antibodies (C). The COX4-twinStrep-GFP (green arrow), COX4-twinStrep-NifB*_Av_* (blue arrow), COX4-twinStrep-NifB*_Mi_* (red arrow) proteins are highlighted. Non-specific bands detected using the Strep-tag antibodies (white stars) co-migrated with Rubisco (B). Non-specific bands detected with NifB*_Av_* antibodies (black stars) are also indicated (C). Short (s.e.) and long (l.e.) film exposures of the Strep-tag antibody probed membrane are shown (B). Ponceau staining of the NifB*_Av_* antibody probed membrane is shown as loading control (C).

Both COX4-twin-Strep-NifB*_Av_* and COX4-twin-Strep-NifB*_Mi_* were readily detected in total protein extracts of *A. tumefaciens* infiltrated *N. benthamiana* leaves (**Figure 8A**). To test the solubility of the expressed NifB proteins, total protein extracts were separated in soluble fractions and pellet associated fractions. COX4-twin-Strep-NifB*_Mi_* was detected exclusively in the soluble fraction, even upon prolonged exposure (**Figure 8B**). On the contrary, COX4-twin-Strep-NifB*_Av_* was more difficult to detect using the Strep-tag II antibody, and appeared to be in the non-soluble fraction. To verify the identity of the NifB*_Av_* protein detected by the Strep-tag II antibody we used NifB*_Av_* specific antibody, which confirmed that COX4-twin-Strep-NifB*_Av_* was mainly present in the pellet associated fraction (**Figure 8C**).

In summary, we show that mitochondria targeting using SU9 and COX4 resulted in expression of both NifB*_Av_* and NifB*_Mi_* in leaves of *N. benthamiana*. Leader sequence processing of all proteins appeared efficient and correct, as only one band of the expected size was detected. Similar to yeast, the NifB*_Mi_* protein was more soluble than the corresponding NifB*_Av_* variant in *N. benthamiana*.

## Discussion

Expression of functional NifB is absolutely required to engineer nitrogenase in eukaryotic organisms (e.g., plants). NifB catalyzes the formation of NifB-co, a unique [Fe–S] cluster intermediate in the biosynthesis of FeMo-co of nitrogenase (Shah et al., 1994; Curatti et al., 2006; Wiig et al., 2012; Guo et al., 2016). All diazotrophs carry at least one *nifB* gene (Dos Santos et al., 2012), and it is not likely that NifB-co can be produced by any other enzyme of plant origin (Vicente and Dean, 2017). As NifB-co also serves as precursor to the FeV-co of V-nitrogenase and the FeFe-co of Fe-only nitrogenase, it is required for all biological nitrogen fixation activity in nature (Bishop and Joerger, 1990; Curatti et al., 2007). Therefore, finding NifB proteins able to function in eukaryotic cells is of utmost importance.

In this work, we investigated eukaryotic expression and functionality of NifB proteins from two evolutionary distant organisms, the γ-proteobacterium *A. vinelandii* and the methanogen *M. infernus*. These NifB proteins differ in domain composition, quaternary structure, optimum temperature and stability, but both contain the same complement of [Fe–S] clusters, catalyze the SAM-radical dependent formation of NifB-co (Curatti et al., 2006; Wilcoxen et al., 2016), and have been shown to support FeMo-co biosynthesis *in vivo* (Arragain et al., submitted manuscript).

As most Nif proteins, NifB is extremely O_2_-labile. Expressed NifB variants were therefore targeted to mitochondria for respiratory protection (Lopez-Torrejon et al., 2016). Mitochondria targeting in yeast was achieved using the SU9 leader sequence, which had proved efficient for Nif protein targeting and processing in a previous study (Burén et al., 2017). Expression of the NifB variants in *S. cerevisiae* was coordinated with expression of *A. vinelandii* NifU, NifS and FdxN, as these proteins are important for the assembly of NifB [Fe–S] clusters and for NifB functionality (Jiménez-Vicente et al., 2014; Zhao et al., 2007). Both NifB variants appeared mostly insoluble and the major protein pools were pelleting together with the cellular debris of the broken cells. The accumulation insoluble NifB might be a result of overexpression or NifB hydrophobicity. It is intriguing to note that overexpression of NifB proteins often result in insoluble aggregates and that purification of NifB from different organisms, as well as purification of NifB-co itself, requires detergents (Curatti et al., 2006; Echavarri-Erasun et al., 2014; Shah et al., 1994).

The two NifB variants were also expressed and targeted to mitochondria of *N. benthamiana* leaf cells. Although the extraction methods and buffers used to prepare protein extracts were slightly different in yeast and tobacco, the results obtained from both systems were similar, i.e. expression levels were slightly higher and solubility was significantly better for the *M. infernus* NifB. Intriguingly, solubility overall appeared better in tobacco than in yeast, perhaps due to lower expression levels from the 35S promoter compared to the very strong GAL promoters. Mitochondria targeting was achieved both with SU9 and COX4 leader sequences, expanding the synthetic toolbox for Nif expression in plants. The identical migration in SDS-PAGE of each set of yeast- and tobacco-expressed NifB variants suggests that all of them underwent correct processing, which in the case of yeast SU9-NifB*_Mi_* was demonstrated to occur at the predicted peptide bond between tyrosine and serine residues.

Taking advantage of the heat-resistant properties of *M. infernus* NifB, a protocol to enrich levels of the protein in the soluble fraction of yeast cell-free extracts was developed permitting further purification and biochemical analysis. Pure yNifB*_Mi_* preparations exhibited properties characteristic of bacteria-purified NifB proteins (Curatti et al., 2006; Wilcoxen et al., 2016), including color, Fe and S content, and UV-vis spectra changes upon [Fe–S] cluster reconstitution and reduction. Importantly, yNifB*_Mi_* showed activities similar to the NifB*_Av_* protein (purified from *A. vinelandii* cells) when used to complement *ΔnifB A. vinelandii* extracts in FeMo-co synthesis assays.

Whether or not NifB proteins were functional *in vivo* in *S. cerevisiae* is not known, as we have not yet been able to establish an *in vivo* assay for NifB activity. In this regard, we tested whether yNifB*_Mi_* in the heat-treated extract was functional without reconstitution, but failed to detect activity, indicating that yNifB*_Mi_* has low or no activity prior to [Fe–S] cluster reconstitution, or that some of its [Fe–S] clusters are lost during yeast cell lysis and/or heat-treatment. Importantly, addition of NifB-co to UW140 extract, in the presence or absence of yeast cell-free extract, resulted in identical activity, suggesting that the heat-treated cell-free extract did not inhibit the *A. vinelandii* nitrogenase activity and that lack of detectable yNifB*_Mi_* activity was not due to inhibition of the yeast extract *per se* (data not shown).

We recently used a synthetic biology approach to assemble a yeast library for the expression of nine *A. vinelandii nif* genes (*nifHDKUSMBEN*), where the gene products were targeted to the yeast mitochondria using distinct mitochondria leader sequences (Burén et al., 2017). That work highlighted that expression levels (using distinct promoter/terminator combinations) and mitochondria signals in many cases need to be empirically tested for each gene. We also learned that two isoforms of the NifD polypeptide accumulated in the yeast mitochondria, one of which was the result of N-terminal degradation of the full-length NifD. A similar result could be observed when NifD was expressed in leaf cells of tobacco (Allen et al., 2017), where their NifD degradation isoform showed a SDS-PAGE migration very similar to ours. A plausible explanation to this NifD degradation could be instability of NifDK precursors, as stability of the NifDK tetramer is improved with protein maturation and nitrogenase co-factor (FeMo-co) insertion. In this regard, expression of functional NifB will stabilize NifDK and help increase its levels.

In summary, we have purified NifB protein expressed in a eukaryotic cell background. Following NifH and NifU (Lopez-Torrejon et al., 2016), to our knowledge this is the third Nif protein that has been purified with specific *in vitro* activity. Using both yeast and tobacco as expression hosts, we observed that the monomeric NifB*_Mi_* (Wilcoxen et al., 2016) was expressed at higher levels, and to a higher extent accumulated as a soluble protein, than the dimeric NifB*_Av_*. This study emphasizes that simpler and more robust Nif protein variants could provide advantages in the ambitious goal of obtaining a functional plant expressed nitrogenase. Importantly, this study confirms that yeast synthetic biology provides a valuable tool in the initial designing and screening process for Nif protein expression and functionality, prior to performing more complex and time-consuming plant-based experiments.

## Materials and Methods

### Generation of Plasmids for Galactose-Induced Yeast Expression

*Escherichia coli* DH5α was used for storage and amplification of yeast expression vectors. *E.coli* was grown at 37°C in Luria-Bertani (LB) medium supplemented with appropriate antibiotics. Yeast optimized coding sequences for *nifU*, *nifS*, *nifB* (*A. vinelandii* and *M. infernus*) and *fdxN* with in-frame su9 leader sequences (Westermann and Neupert, 2000) were generated by GenScript, or by overlapping PCR reactions as specified below, and cloned into pESC vectors (Agilent Technologies) using standard techniques. *su9-nifU* and *su9-nifS* were cloned into pESC-URA using *BamH*I/*Hind*III and *EcoR*I/*Bgl*II, respectively, generating pN2GLT4. *su9-fdxN* and *su9-His*_10_-*nifB_Av_* were cloned into pESC-TRP using *Not*I/*Cla*I and *BamH*I/*Sal*I, respectively, generating pN2GLT18. *su9-nifB_Av_-His*_10_ and *su9-nifB_Mi_-His*_10_ were created using overlapping PCR, to add su9 and His_10_ at the 5′- and 3′-termini of *nifB_Av_* and *nifB_Mi_*. Primers used for generating *su9-nifB_Av_-His*_10_ were 5′-ATTTTCGGTTTGTATTACTTC-3′ and 5′-CATGGAAGAGTAGGCGC-3′ (using pN2GLT18 as template), 5′-GCGCCTACTCTTCCATGGAATTGTCTGTTTTGGGT-3′ and 5′-ATGATGGTGGTGGTGATGATGATGAGCCTTAGCTTGCAAC-3′ (using pN2GLT18 as template), 5′-ATCACCACCACCATCATCACCATTAAGTCGACATGGAACA-3′ and 5′-GTACACGCGTCTGTACAGAA-3′ (using pN2GLT18 as template), to amplify su9, *nifB_Av_* and His_10_, respectively. 5′-ATTTTCGGTTTGTATTACTTC-3′ and 5′-GTACACGCGTCTGTACAGAA-3′ were used for the overlapping PCR reaction. Primers used for generating *su9-nifB_Mi_-His*_10_ were 5′-ATTTTCGGTTTGTATTACTTC-3′ and 5′-CATGGAAGAGTAGGCGC-3′ (using pN2GLT18 as template), 5′-GCGCCTACTCTTCCATGGAGAAAATGTCTAAATTT-3′ and 5′-ATGATGGTGGTGGTGATGATGATGGTGTGAGAAATGCTTC-3′ (using *nifB_Mi_* as template), 5′-ATCACCACCACCATCATCACCATTAAGTCGACATGGAACA-3′ and 5′-GTACACGCGTCTGTACAGAA-3′ (using pN2GLT18 as template), to amplify su9, *nifB_Mi_* and His_10_, respectively. 5′-ATTTTCGGTTTGTATTACTTC-3′ and 5′-GTACACGCGTCTGTACAGAA-3′ were used for the overlapping PCR reaction. *su9-nifB_Av_-His*_10_ and *su9-nifB_Mi_-His*_10_ were cloned into pN2GLT18, replacing *su9-His*_10_-*nifB_Av_* using *BamH*I/*Sal*I, and generating pN2SB22 and pN2SB24, respectively. *su9-fdxN-HA* was created using overlapping PCR, to add HA at the 3′-terminus of *su9-fdxN*. Primers used for generating *su9-fdxN-HA* were 5′-GGTGGTAATGCCATGTAATATG-3′ and 5′-GCATAATCTGGAACATCATATGGATACCTTGCCTGTATTT-3′ (using pN2SB22 as template), 5′-GATGTTCCAGATTATGCTTAAGAGCTCTTAATTAACAATT-3′ and 5′-AAAGTTTAAACCGCATCAGGAAATTGTAA-3′ (using pN2SB22 as template), to amplify *su9-fdxN* and HA, respectively. 5′-GGTGGTAATGCCATGTAATATG-3′ and 5′-AAAGTTTAAACCGCATCAGGAAATTGTAA-3′ were used for the overlapping PCR reaction. *su9-fdxN-HA* was cloned into pN2SB24, replacing *su9-fdxN* using *Not*I/*Pac*I, generating pN2SB39. To make pN2GLT18 (*su9-fdxN* and *su9-His*_10_-*nifB_Av_*) compatible with transformation into prototrophic *S. cerevisiae* CEN.PK113-7D clone DOE56, the LEU2 auxotrophic marker was replaced with the hygromycin marker *hphMX4* (Burén et al., 2017), generating pN2SB15. DNA and protein sequences of all constructs are listed in Supplementary Figure S2.

### Generation of Yeast Strains, Growth, and Protein Expression

*Saccharomyces cerevisiae* W303-1a (*MAT*a *leu2-3,112 trp1-1 can1-100 ura3-1 ade2-1 his3-11,15*) was the host strain for expression vectors pN2GLT4 and pN2SB22 (to generate strain SB09Y), pN2GLT4 and pN2SB24 (to generate strain SB10Y), and pN2GLT4 and pN2SB39 (to generate strain SB12Y). CEN.PK113-7D (*MATa URA3 TRP1 LEU2 HIS3 MAL2-8c SUC2*) strain DOE56 (having constitutive expression of mitochondria targeted NifU and NifS) (Burén et al., 2017) was the host strain for expression vector pN2SB15 (to generate strain SB03Y). Yeast transformations were carried out according to the lithium acetate method (Gietz and Schiestl, 2007).

*Saccharomyces cerevisiae* were grown in flasks at 28°C and 200 rpm in synthetic drop-out (SD) medium (1.9 g/l yeast nitrogen base, 5 g/l ammonium sulfate, 20 g/l glucose, and Kaiser drop-out mixture (Kaiser et al., 1994) (SC -His-Leu-Trp-Ura, FORMEDIUM) supplemented with 20 mg/l adenine and 40 mg/l tryptophan, 40 mg/l histidine, 20 mg/l uracil, 60 mg/l leucine, depending on auxotrophic requirements). Plasmid for the inducible expression of SU9-FdxN and SU9-His_10_-NifB*_Av_* in transformed DOE56 (SB03Y) was maintained by supplementing the inoculum growth media with 300 μg/l hygromycin. Galactose induction for small-scale protein extracts was performed in the above-described SD medium in which glucose was replaced by 20 g/l galactose, and additionally supplemented with 0.1% yeast extract and 1% peptone. Total yeast protein extracts to verify protein expression were performed using mild alkali treatment (Kushnirov, 2000). Similar loading on SDS-PAGE experiments was obtained by preparing samples according to optical density, and was confirmed by using either Commassie staining of polyacrylamide gels or Ponceau staining of nitrocellulose membranes. Additionally, immunoblotting with antibodies against tubulin was used as control of gel loading and sample precipitation.

Cultures for yeast expressed NifB purifications were grown following a previously reported procedures (Lopez-Torrejon et al., 2016; Burén et al., 2017), in a 4-L fermenter (BIO-STAT). Cultures were grown at 30°C in selective SD-medium for 16 h, followed by 8 h in rich medium (0.5% yeast extract, 0.5% bactopeptone, 0.5% bactotryptone, 2.5% glucose), supplemented with 25 mg/L ammonium iron(III) citrate, 1.25 mM magnesium sulfate, 1.5 mM calcium chloride, and trace element and vitamin solutions (Lopez-Torrejon et al., 2016). Finally, protein expression was induced by addition of 2.5% galactose for 16 h. The pH was automatically maintained around 5 using 0.8 M ammonium hydroxide. Air flow was maintained at 0.75 liter air/min per liter of culture, at 300 rpm. Dissolved oxygen dropped to zero (as measured by oxygen sensor, Mettler Toledo) before addition of galactose, and remained at zero during the rest of the process.

### Reverse Transcription and Quantitative Real-time Polymerase Chain Reactions

Total yeast RNA was extracted from 25 ml cultures 6 h following galactose induction. Briefly, the cells were harvested by centrifugation (10 min at 3,000 x *g*), washed once in Milli-Q water and then resuspended in 1 ml TRIzol reagent (ThermoFisher Scientific). Cells were broken in 2 ml screw-cap tubes using 0.5 mm glass beads (BioSpec Products) in a mixer mill (Retsch MM300) operating at 30 Hz, in 5 cycles of 1 min at 4°C. Two hundred μl chloroform was added to the lysate, vortexed for 15 sec and incubated 5 min at room temperature. Samples were then centrifuged at full speed for 5 min at 4°C. The supernatant was transferred to new tube and re-extracted with 400 μl chloroform. The supernatant containing RNA was precipitated with 1 volume isopropanol at -20°C for 20 minutes and then pelleted by centrifugation at full speed for 5 min at 4°C. The pellet was washed twice in 1 ml 70% ethanol and finally resuspended in nuclease free water. RNA concentration was measured in a nanodrop apparatus and quality was analysed by agarose gel electrophoresis. RNA was then treated with DNAse to remove eventual DNA contamination (TURBO DNA-free Kit, AM1907, Ambion). Absence of DNA was verified using polymerase chain reaction (PCR) (KAPA2G Fast HotStart ReadyMix, B4KK5609, KAPA2G). Four μg RNA was used for cDNA synthesis (High-Capacity cDNA Reverse transcription Kit, 4374966, Applied Biosystems). The presence of *fdxN* cDNA was verified by reverse transcription PCR (RT-PCR) using 4 distinct *fdxN* primer combinations (A, 5′-TGTGAACTGCTGGGCATGTG-3′ and 5′-TCTCCATCACACTCGGTGCAT-3′; B, 5′-TGCACCGAGTGTGATGGAGA-3′ and 5′-TGCCTCAGCCAATCTTTCAGGT-3′; C, 5′-ACCGAGTGTGATGGAGACTAT-3′ and 5′-GTAAGTGAACCAGGTGGGTTAG-3′; D, 5′-CCTTGGCAGGTCCTCATTT-3′ and 5′-TAGCACCTTCAACTGGACAAATA-3′). Primers targeting housekeeping genes (*alg9*, 5′-CACGGATAGTGGCTTTGGTGAACAATTAC-3′ and 5′-TATGATTATCTGGCAGCAGGAAAGAACTTGGG-3′; *rdn18*, 5′-AACTCACCAGGTCCAGACACAATAAGG-3′ and 5′-AAGGTCTCGTTCGTTATCGCAATTAAGC-3′) were selected based on Teste and colleagues (Teste et al., 2009). Quantitative real-time PCR (qPCR) (KAPA SYBR FAST Universal qPCR Kit, KK4601, KAPA2G) was performed using two *fdxN* primer combinations in addition to primers targeting the housekeeping genes, using an Eco Real-Time PCR System (Illumina) following user instructions.

### Solubility of Yeast-Expressed NifB

*Saccharomyces cerevisiae* cells expressing yNifB*_Av_* and yNifB*_Mi_* were resuspended in 5 volumes of lysis buffer (100 mM Tris-HCl, 400 mM NaCl, 5 mM β-mercaptoethanol (β-ME), 1 mM phenyl-methylsulfonyl fluoride (PMSF)), at pH 7 or 8 with 10% or 30% glycerol. Cells were broken in 2 ml tubes using 0.5 mm glass beads (BioSpec Products) in a mixer mill (Retsch MM300) operating at 30 Hz in 3 cycles of 1 min at 4°C. Lysates were incubated at room temperature (RT), or heated at 5°C temperature intervals from 40°C to 75°C, for 30 min. The supernatant after 20 min centrifugation at 20,000 x *g* and 4°C containing soluble proteins was analyzed by SDS-PAGE and immunoblot analysis.

### Preparation of Yeast Anaerobic Cell-Free Extracts and NifB Purifications

*Saccharomyces cerevisiae* cells expressing yNifB*_Av_* and yNifB*_Mi_* were resuspended in anaerobic lysis buffer (100 mM Tris-HCl pH 8.0, 400 mM NaCl, 10% glycerol) supplemented with 2 mM dithionite (DTH), 5 mM β-ME, 1 mM PMSF, 1 μg/ml leupeptin and 5 μg/ml DNAse I. The cells were lysed in an Emulsiflex-C5 homogenizer (Avestin Inc.) at 25,000 lb per square inch. Cell-free extracts (CFE) were obtained after heat-treatment in a water bath (yNifB*_Mi_* only, 65°C for 30 min), removal of cell debris and precipitated yeast proteins by centrifugation (50,000 x *g* for 1 h at 4°C) and filtration through a 0.2 μM pore size filter (Nalgene Rapid-Flow, Thermo Scientific). All procedures were performed under anaerobic conditions.

*Saccharomyces cerevisiae* cells expressing SU9-His_10_-NifB*_Av_* were lysed as described above, in buffer with detergents (50 mM Tris-HCl pH 8, 200 mM KCl, 10% glycerol, 5 mM β-ME, 0.05% n-dodecyl-β-D-maltopyranoside, 0.1% triton X-100 and 0.1% Tween 20) as previously described for purification of NifB*_Mi_* from *E. coli* (Wilcoxen et al., 2016).

His-tagged yNifB*_Mi_* was purified by Co^2+^ affinity chromatography under anaerobic conditions (<0.1 ppm of O_2_) using an AKTA Prime FPLC system (GE Healthcare) inside a glovebox (MBraun). All buffers were previously made anaerobic by sparging with N_2_. Before loading the affinity column, the cell-free extract was diluted to reach 50 mM Tris-HCl, while maintaining other buffer components. Typically, anaerobic cell-free extract from 100 g of cell paste was loaded at 2 ml/min onto a column filled with 5 ml of IMAC resin (GE Healthcare) equilibrated with buffer A (50 mM Tris-HCl pH 8, 400 mM NaCl, 10% glycerol, 2 mM DTH, 5 mM β-ME) and washed with four successive washes of buffer A supplemented with 0, 10, 40 and 100 mM imidazole (10-15 column volumes per wash), respectively. Bound protein was eluted in two steps, with buffer A containing 200 and 500 mM imidazole, respectively. Eluted fractions showing the desired purity were pooled and concentrated using a 100 kDa cutoff pore centrifugal membrane device (Amicon Ultra-15, Millipore), and then desalted in PD10 columns (GE Healthcare) equilibrated with buffer A. Pure yNifB*_Mi_* was frozen and stored in liquid N_2_.

### *In Vitro* Reconstitution of yNifB*_Mi_* Fe-S Clusters, UV-Visible Spectroscopy, N-terminal Sequencing and Protein Methods

*In vitro* reconstitution of purified yNifB*_Mi_* was performed as previously described with modifications (Curatti et al., 2006). Pure yNifB*_Mi_* stored in buffer A was buffer-exchanged to buffer B (50 mM Tris-HCl pH 8, 400 mM NaCl, 10% glycerol, 5 mM β-ME) by using a PD10 column to recover “as isolated” protein. The desalted sample (20 μM NifB monomer) was incubated with 10 mM DTT at room temperature inside a glovebox (MBraun) for 10 min. (NH_4_)_2_Fe(SO_4_)_2_ and Na_2_S were then added at 20-fold molar excess ratio and incubated at 35°C overnight. yNifB*_Mi_* was again desalted in buffer B to recover “reconstituted” protein. As isolated and reconstituted proteins were used for colorimetric Fe (Fish, 1988) and S (Beinert, 1983) determination, *in vitro* FeMo-co synthesis and nitrogenase activity assays, and UV-visible spectroscopy. UV-visible absorption spectra were recorded under anaerobic conditions in septum-sealed cuvettes using a Shimadzu UV-2600 spectrophotometer. When indicated, 5 mM DTH was added to reconstituted yNifB*_Mi_*. UV-visible absorption spectra were recorded against buffer B as baseline. Absorbance at 800 nm was subtracted and spectra were then normalized to 279 nm. The N-terminal amino acid sequence of purified yNifB*_Mi_* was determined by Edman degradation (Proteome Factory AG). Protein concentrations were measured using the BCA protein assay (PIERCE). NifB samples were pre-treated with iodoacetamide before performing the BCA assay to eliminate the interfering effect of DTH (Hill and Straka, 1988).

### *In Vitro* Synthesis of FeMo-co and Nitrogenase Reconstitution Assay

*In vitro* yNifB*_Mi_* dependent FeMo-co synthesis and nitrogenase reconstitution reactions were performed in 9-ml serum vials sealed with serum stoppers (Curatti et al., 2006). Complete reactions contained 17.5 μM Na_2_MoO_4_, 175 μM homocitrate, 1.75 mM (NH_4_)_2_FeSO_4_, 1.75 mM Na_2_S, 880 μM SAM, 1.23 mM ATP, 18 mM phosphocreatine, 2.2 mM MgCl_2_, 3 mM DTH, 40 μg/ml creatine phosphokinase, 2.2 μM NifH (dimer), 2.9 mg/ml UW140 (*A. vinelandii ΔnifB*) proteins, 5 μM (or 0-10 μM titration) reconstituted yNifB*_Mi_* (monomer) in 22 mM Tris-HCl (pH 7.5). The reactions (total volume of 500 μl) were incubated at 30°C for 35 min to allow for FeMo-co synthesis and insertion reactions. NifB-co-dependent *in vitro* FeMo-co synthesis assays were performed using 2 μM NifB-co isolated from *K. oxytoca* (Shah et al., 1994). Following *in vitro* synthesis of FeMo-co, activation of apo-MoFe nitrogenase present in UW140 extract was analyzed following addition of excess NifH and ATP-regenerating mixture (total volume 1 ml) by acetylene reduction assay at 30°C for 30 min following standard procedures (Shah and Brill, 1973). Positive control reactions for acetylene reduction were carried out with pure preparations of *A. vinelandii* Fe protein and MoFe protein incubated with ATP-regenerating mixture at 30°C during 30 min.

### Generation of Plant Expression Vectors and Protein Expression in Leaves of *N. benthamiana*

*Escherichia coli* DH5α was used for storage and amplification of plant expression vectors. *E. coli* was grown at 37°C in LB medium supplemented with appropriate antibiotics. *su9-nifB_Av_-His*_10_ and *su9-nifB_Mi_-His*_10_ were PCR amplified using primers 5′-AAAAGGATCCAATGGCCTCCACTCGTGTCCTCG-3′ and 5′-TTTTCACGTGTTAATGGTGATGATGGTGGTG-3′, with pN2SB22 and pN2SB24 as templates, respectively. *su9-nifB_Av_-His*_10_ and *su9-nifB_Mi_-His*_10_ were digested with *BamH*I and *Pml*I, and inserted into pGFPGUSPlus vector (Vickers et al., 2007) (Addgene plasmid #64401) digested with *Bgl*II and *Pml*I, replacing GUS and generating pN2XJ13 (*su9-nifB_Av_-His*_10_) and pN2XJ14 (*su9-nifB_Mi_-His*_10_), respectively. *su9-nifB_Av_* was PCR amplified using primers 5′-AAAAGCTAGCATGGCCTCCACTCGTGTCCTCG-3′ and 5′-TTTTGCTAGCGCCTTAGCTTGCAACAAAGC-3′, with pN2SB22 as template. *su9-nifB_Av_* was digested with *Nhe*I and inserted into pGFPGUSPlus vector digested with *Xba*I, generating pN2XJ15 for expression of *su9-nifB_Av_-gfp. su9-nifB_Mi_* was PCR amplified using primers 5′-AAAAGCTAGCATGGCCTCCACTCGTGTCCTCG-3′ and 5′-TTTTGCTAGCGCGTGTGAGAAATGCTTCAAGTCG-3′, with pN2SB24 as template. *su9-nifB_Mi_* was digested with *Nhe*I and inserted into pGFPGUSPlus vector digested with *Xba*I, generating pN2XJ16 for expression of *su9-nifB_Mi_-gfp*. DNA sequence encoding the enhanced 35S promoter and an in-frame fusion of the *cox4* mitochondria leader sequence (Köhler et al., 1997) with the 28 amino acid Twin-Strep-tag was generated by ThermoFisher. The E35S-cox4-twinStrep DNA sequence was flanked by *Hind*III and *Bgl*II, with a *BamH*I site additionally added 5′ of the *Bgl*II site. E35S-cox4-twinStrep was digested with *Hind*III and *Bgl*II, and inserted into pGFPGUSPlus vector also digested with *Hind*III and *Bgl*II, to generate pN2SB41. DNA sequence encoding *egfp* was PCR amplified using primers 5′-AAAAAGGATCCATGGTGAGCAAGGGCGA-3′ and 5′-AAAAAGGTCACCTTACTTGTACAGCTCGTCCATG-3′, and pGFPGUSPlus as template. *egfp* was digested with *BamH*I and *BstE*II, and inserted into pN2SB41 also digested with *BamH*I and *BstE*II, creating pN2XJ17. pN2XJ17 was digested with *Pst*I to remove the non-targeted EGFP, to generate pN2XJ19 (*cox4-twinStrep-gfp*). DNA sequences encoding *nifB_Av_* and *nifB_Mi_*, flanked by *BamH*I and *BstE*II, were generated by ThermoFisher. *nifB_Av_* and *nifB_Mi_* were digested with *BamH*I and *BstE*II, and inserted into pN2SB41 also digested with *BamH*I and *BstE*II, to generate pN2XJ20 (*cox4-twinStrep-nifB_Av_*) and pN2XJ21 (*cox4-twinStrep-nifB_Mi_*). DNA and protein sequences of all constructs are listed in Supplementary Figure S8.

*Agrobacterium tumefaciens* strain GV3101(pMP90) was transformed with plasmids pN2XJ13, pN2XJ14, pN2XJ15, pN2XJ16, pN2XJ19, pN2XJ20, pN2XJ21 and the silencing suppressor p19 (Huang et al., 2009). The pDCL-mito-mRFP1 mitochondria marker (Mito-RFP) in *A. tumefaciens* strain C58 (Candat et al., 2014) was kindly provided by Prof. Macherel and Prof. Logan at the Angers University (France). *A. tumefaciens* mediated infiltration of *N. benthamiana* leaves was essentially performed as described by Leuzinger and colleagues (Leuzinger et al., 2013). Three to four days post infiltration, plant tissue was used for protein extraction or confocal microscopy.

Protein extracts were prepared from infiltrated *N. benthamiana* leaf tissue in lysis buffer (100 mM Tris-HCl pH 8, 150 mM NaCl, 10 mM MgCl_2_, 0.2% NP-40, 5% glycerol, 5 mM β-ME and 5 mM ethylenediaminetetraacetic acid (EDTA)). Two hours before use, 5% polyvinylpolypyrrolidone (PVPP) was added to lysis buffer and, just before use, 1 mM PMSF, 1 μg/ml leupeptin and 1x protease inhibitor cocktail (P8215, Sigma) were added. Extraction was performed at a 2:1 ratio of buffer to tissue. Ten leaf discs of 5 mm diameter each (approximate weight of 200 mg) were added to a 2-ml Eppendorf tube containing a 7-mm diameter steel ball. Tubes were kept in liquid N_2_ until use. Leaf tissue was broken using mixer mill (Retsch MM300) operating at 30 Hz for 1 min at 4°C. The dry tissue powder was supplemented with 400 μl lysis buffer and mixed for another 1 min at 30 Hz and 4°C. The broken tissue in lysis buffer was further incubated on an orbital shaker for 30 min at 4°C. One hundred μl extract were added to 100 μl 2x Laemmli buffer (2xLB) and heated for 10 min at 95°C to obtain the “total extract”. The rest of the extract was centrifuged at 20,000 x *g* for 30 min at 4°C to separate pellet from supernatant. The supernatant “soluble extract (S)” was mixed with 2xLB and heated for 10 min at 95°C. The pellet (P) was resuspended in 1 ml lysis buffer (no additional PVPP added) and centrifuged at 20,000 x *g* for 10 min at 4°C. Finally, the pellet was resuspended in 800 μl 2xLB and heated for 10 min at 95°C. Ten μl of each fraction were used for SDS-PAGE and immunoblot analysis. Similar sample loading on SDS-PAGE lanes was assessed either by Commassie staining of polyacrylamide gels, by Ponceau staining of transferred nitrocellulose membranes, or by immunoblotting with antibodies against Rubisco.

### Confocal Microscopy of *N. benthamiana* Leaf Tissue

Subcellular localization of fluorescent protein tagged proteins was examined in leaves of *A. tumefaciens* infiltrated *N. benthamiana* using a Leica TCS SP8 laser scanning confocal microscope with a 40x/1.10 water immersion objective equipped with LAS X software (Leica). EGFP, RFP, and chlorophyll were excited with 488-, 561-, or 638-nm laser lines, respectively, with an emission band of 500 to 537 nm for EGFP detection, 585 to 620 nm for RFP detection, and 652 to 727 nm for chlorophyll autofluorescence. EGFP and chlorophyll was recorded simultaneously, while RFP was detected in a separate scan. Laser intensity and gain was maintained during each experiment. For each experiment, specificity of the recorded signals was verified using single transformed cells.

### Antibodies

Antibodies used for immunoblotting in this study were as follows: polyclonal antibodies detecting NifU*_Av_*, NifS*_Av_*, NifB*_Av_* and NifB*_Mi_* were raised against purified preparations of the corresponding *A. vinelandii* or *M. infernus* proteins. Rubisco specific antibodies were a kind gift from Prof. Göran Samuelsson, Umeå University. His-tag (H-3, sc-8036, Santa Cruz), HA-tag (3F10, 12013819001, Roche), GFP (B-2, sc-9996, Santa Cruz), Strep-tag II (StrepMAB-Classic, 2-1507-001, IBA Lifesciences) specific antibodies are commercially available.

## Author Contributions

SB, XJ, GL-T, and CE-E carried out the experimental work. SB, XJ, GL-T, CE-E and LR contributed to experimental design and data analysis. SB and LR wrote the paper.

## Conflict of Interest Statement

The authors declare that the research was conducted in the absence of any commercial or financial relationships that could be construed as a potential conflict of interest.
