# Supplementary material for: Purification and In Vitro Activity of Mitochondria Targeted Nitrogenase Cofactor Maturase NifB
Source: Front Plant Sci. 2017 Sep 12;8:1567. doi: 10.3389/fpls.2017.01567 (PMC5601070; doi:10.3389/fpls.2017.01567)
Supplement: Supplementary file 1 [file Presentation_1.pdf]

Supplementary Material

Purification and in vitro activity of mitochondria targeted nitrogenase cofactor maturase NifB

Stefan Burén, Xi Jiang, Gema Lopez-Torrejón, Carlos Echavarri-Erasun, and Luis M. Rubio*

*** Correspondence:** Luis M. Rubio: lm.rubio@upm.es

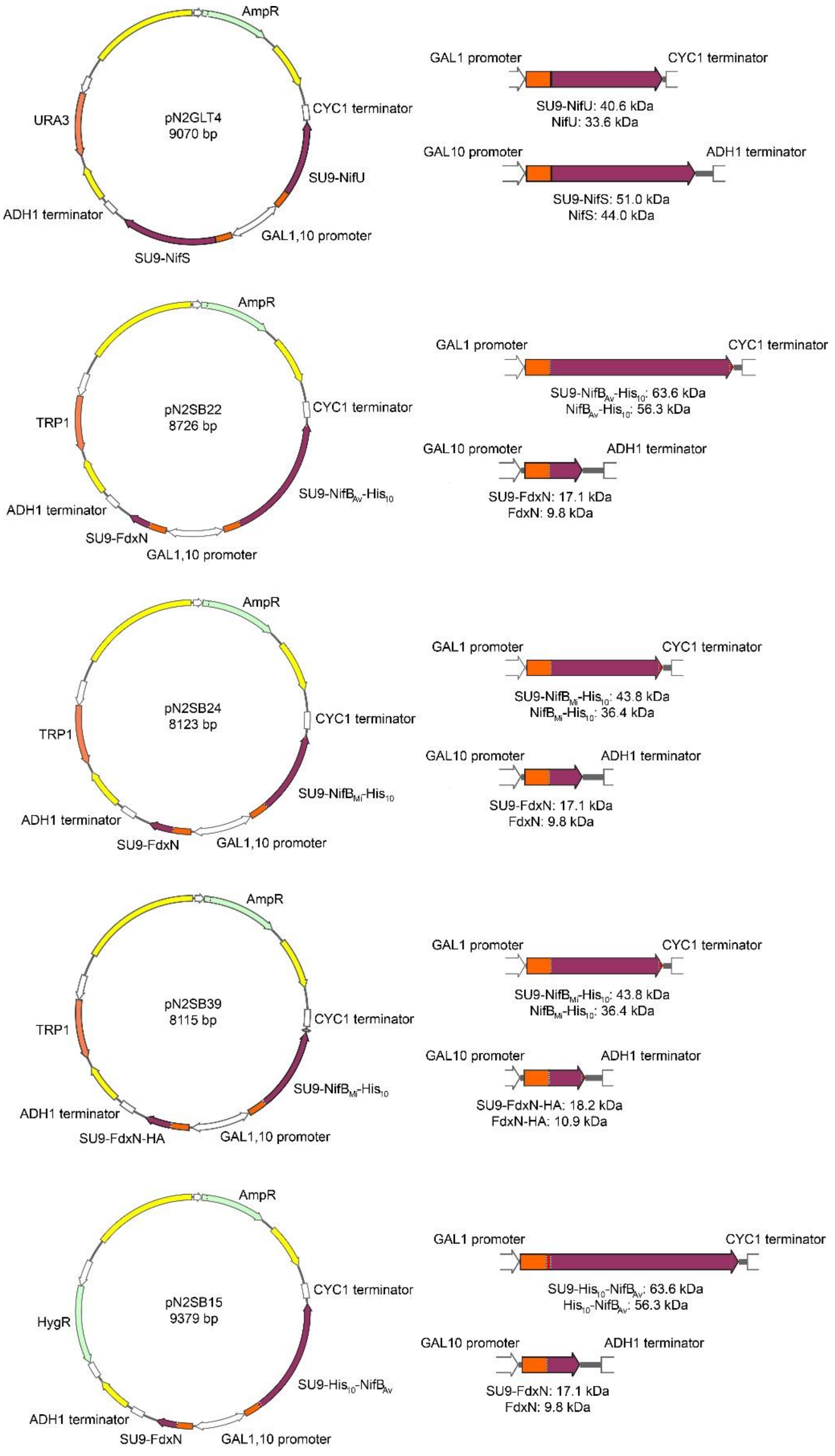

SUPPLEMENTARY FIGURE 1. Yeast expression vectors. Schematic overview of yeast expression vectors constructed in this study. See Supplementary Figure 2 for detailed information about the codon-optimized *nif* sequences.

SU9-**NifU**

1 M A S T R V L A S R L A S Q M
1 ATGGCCTCCACTCGTGTCCTCGCCTCTCGCCTGGCCTCCCAGATG

16 A A S A K V A R P A V R V A Q
46 GCTGCTTCCGCCAAGGTTGCCCGCCCTGCTGTCCGCGTTGCTCAG

31 V S K R T I Q T G S P L Q T L
91 GTCAGCAAGCGCACCATCCAGACTGGCTCCCCCCTCCAGACCCTC

46 K R T Q M T S I V N A T T R Q
136 AAGCGCACCCAGATGACCTCCATCGTCAACGCCACCACCCGCCAG

61 A F Q K R A Y S S R P **W D Y S**
181 GCTTTCCAGAAGCGCGCCTACTCTTCCAGGCCTTGGGACTACTCT

76 **E K V K E H F Y N P K N A G A**
226 GAAAAGGTTAAGGAACATTTCTACAATCCAAAGAACGCCGGTGCT

91 **V E G A N A I G D V G S L S C**
271 GTAGAAGGTGCAAACGCCATTGGTGACGTTGGTTCATTATCCTGT

106 **G D A L R L T L K V D P E T D**
316 GGTGACGCTTTGAGATTAACATTGAAAGTTGACCCTGAAACCGAT

121 **V I L D A G F Q T F G C G S A**
361 GTCATCTTGGACGCAGGTTTTCAAACTTTCGGTTGCGGTTCTGCT

136 **I A S S S A L T E M V K G L T**
406 ATTGCATCTTCATCCGCTTTGACTGAAATGGTTAAGGGTTTGACA

151 **L D E A L K I S N Q D I A D Y**
451 TTGGATGAAGCATTGAAAATCTCAAACCAAGATATCGCTGACTAT

166 **L D G L P P E K M H C S V M G**
496 TTGGATGGTTTGCCACCTGAAAAGATGCATTGTTCCGTCATGGGT

181 **R E A L Q A A V A N Y R G E T**
541 AGAGAAGCCTTACAAGCTGCAGTAGCTAACTACAGAGGTGAAACC

196 **I E D D H E E G A L I C K C F**
586 ATTGAAGATGACCACGAAGAAGGTGCATTGATATGTAAATGCTTT

211 **A V D E V M V R D T I R A N K**
631 GCCGTTGATGAAGTTATGGTCAGAGATACCATAAGAGCAAATAAG

226 **L S T V E D V T N Y T K A G G**
676 TTAAGTACTGTAGAAGATGTTACTAACTACACAAAAGCTGGTGGT

241 **G C S A C H E A I E R V L T E**
721 GGTTGTTCTGCTTGCCATGAAGCAATAGAAAGAGTTTTGACAGAA

256 **E L A A R G E V F V A A P I K**
766 GAATTGGCCGCTAGAGGTGAAGTATTCGTTGCAGCCCCAATTAAA

271 **A K K K V K V L A P E P A P A**
811 GCCAAAAAGAAAGTCAAGGTATTGGCTCCAGAACCTGCCCCAGCT

286 **P V A E A P A A A P K L S N L**
856 CCTGTTGCAGAAGCCCCAGCTGCAGCCCCTAAGTTGTCAAATTTG

301 **Q R I R R I E T V L A A I R P**
901 CAAAGAATTAGAAGAATCGAAACAGTCTTGGCTGCAATAAGACCT

316 **T L Q R D K G D V E L I D V D**
946 ACCTTGCAAAGAGACAAAGGTGACGTCGAATTAATTGATGTAGAC

331 **G K N V Y V K L T G A C T G C**
991 GGTAAAAATGTTTACGTCAAATTGACCGGTGCTTGTACTGGTTGC

346 **Q M A S M T L G G I Q Q R L I**
1036 CAAATGGCATCCATGACATTAGGTGGTATACAACAAAGATTGATC

361 **E E L G E F V K V I P V S A A**
1081 GAAGAATTGGGTGAGTTCGTCAAAGTTATCCCAGTCTCCGCTGCC

376 **A H A Q M E V** *
1126 GCACACGCCCAAATGGAAGTCTGA

SU9-**NifS**

1 M A S T R V L A S R L A S Q M
1 ATGGCCTCCACTCGTGTCCTCGCCTCTCGCCTGGCCTCCCAGATG

16 A A S A K V A R P A V R V A Q
46 GCTGCTTCCGCCAAGGTTGCCCGCCCTGCTGTCCGCGTTGCTCAG

31 V S K R T I Q T G S P L Q T L
91 GTCAGCAAGCGCACCATCCAGACTGGCTCCCCCCTCCAGACCCTC

46 K R T Q M T S I V N A T T R Q
136 AAGCGCACCCAGATGACCTCCATCGTCAACGCCACCACCCGCCAG

61 A F Q K R A Y S S R P **A D V Y**
181 GCTTTCCAGAAGCGCGCCTACTCTTCCAGGCCTGCCGACGTTTAC

76 **L D N N A T T R V D D E I V Q**
226 TTGGATAATAACGCTACTACAAGAGTCGATGACGAAATAGTACAA

91 **A M L P F F T E Q F G N P S S**
271 GCTATGTTGCCATTTTTCACAGAACAATTCGGTAACCCTTCCAGT

106 **L H S F G N Q V G M A L K K A**
316 TTGCATTCCTTCGGTAACCAAGTTGGTATGGCCTTGAAGAAAGCT

121 **R Q S V Q K L L G A E H D S E**
361 AGACAATCTGTCCAAAAATTGTTAGGTGCAGAACACGATTCCGAA

136 **I V F T S C G T E S D S T A I**
406 ATCGTTTTTACCAGTTGTGGTACTGAATCTGACTCAACCGCCATT

151 **L S A L K A Q P E R K T V I T**
451 TTGTCTGCCTTAAAAGCTCAACCAGAAAGAAAGACTGTCATAACC

166 **T V V E H P A V L S L C D Y L**
496 ACTGTTGTCGAACATCCTGCAGTATTGTCTTTATGCGATTATTTG

181 **A S E G Y T V H K L P V D K K**
541 GCCTCAGAAGGTTACACTGTTCATAAGTTACCAGTCGATAAAAAG

196 **G R L D L E H Y A S L L T D D**
586 GGTAGATTGGACTTAGAACACTATGCTTCCTTGTTAACAGATGAC

211 **V A V V S V M W A N N E T G T**
631 GTAGCTGTAGTTAGTGTTATGTGGGCAAATAACGAAACTGGTACA

226 **L F P I E E M A R L A D D A G**
676 TTGTTTCCAATTGAAGAAATGGCAAGATTAGCCGATGACGCTGGT

241 **I M F H T D A V Q A V G K V P**
721 ATAATGTTCCATACTGATGCAGTACAAGCCGTTGGTAAAGTCCCT

256 **I D L K N S S I H M L S L S G**
766 ATAGACTTGAAGAACTCGTCAATCCACATGTTGTCCTTAAGTGGT

271 **H K L H A P K G V G V L Y L R**
811 CATAAATTGCACGCTCCAAAGGGTGTTGGTGTCTTGTACTTAAGA

286 **R G T R F R P L L R G G H Q E**
856 AGAGGTACAAGATTCAGACCTTTGTTAAGAGGTGGTCATCAAGAA

301 **R G R R A G T E N A A S I I G**
901 AGAGGTAGAAGAGCCGGTACTGAAAATGCTGCATCTATTATAGGT

```
 316 L G V A A E R A L Q F M E H E
 946 TTGGGTGTTGCCGCTGAAAGAGCTTTACAATTCATGGAACATGAA

 331 N T E V K R L R D K L E A G I
 991 AACACTGAAGTTAAGAGATTGCGTGATAAGTTAGAAGCAGGTATT

 346 L A V V P H A F V T G D P D N
1036 TTGGCCGTCGTACCACACGCATTTGTTACTGGTGACCCAGACAAT

 361 R L P N T A N I A F E Y I E G
1081 AGATTACCTAACACAGCTAACATCGCATTCGAATACATCGAAGGT

 376 E A I L L L L N K V G I A A S
1126 GAAGCTATCTTGTTGTTGTTGAACAAAGTTGGTATAGCAGCCTCC

 391 S G S A C T S G S L E P S H V
1171 AGTGGTTCTGCTTGTACATCTGGTTCATTGGAACCATCACATGTT

 406 M R A M D I P Y T A A H G T V
1216 ATGAGAGCAATGGATATTCCTTATACAGCTGCACACGGTACTGTT

 421 R F S L S R Y T T E E E I D R
1261 AGATTTTCTTTGAGTAGATACACAACCGAAGAAGAAATTGATAGA

 436 V I R E V P P I V A Q L R K L
1306 GTCATTAGAGAAGTACCACCTATTGTTGCTCAATTGAGAAAATTG

 451 S P Y W S G N G P V E D P G K
1351 TCTCCTTACTGGTCAGGTAATGGTCCTGTTGAAGACCCTGGTAAA

 466 A F A P V Y G *
1396 GCCTTTGCTCCTGTCTATGGTTGA
```

SU9-**FdxN**

```
   1 M A S T R V L A S R L A S Q M
   1 ATGGCCTCCACTCGTGTCCTCGCCTCTCGCCTGGCCTCCCAGATG

  16 A A S A K V A R P A V R V A Q
  46 GCTGCTTCCGCCAAGGTTGCCCGCCCTGCTGTCCGCGTTGCTCAG

  31 V S K R T I Q T G S P L Q T L
  91 GTCAGCAAGCGCACCATCCAGACTGGCTCCCCCCTCCAGACCCTC

  46 K R T Q M T S I V N A T T R Q
 136 AAGCGCACCCAGATGACCTCCATCGTCAACGCCACCACCCGCCAG
```

```
 61 A  F  Q  K  R  A  Y  S  S  M  A  L  K  I  V
181 GCTTTCCAGAAGCGCGCCTACTCTTCCATGGCTCTTAAGATAGTT

 76 E  S  C  V  N  C  W  A  C  V  D  V  C  P  S
226 GAGTCTTGTGTGAACTGCTGGGCATGTGTTGATGTGTGCCCAAGT

 91 E  A  I  S  L  A  G  P  H  F  E  I  S  A  S
271 GAGGCTATATCCTTGGCAGGTCCTCATTTTGAAATTTCTGCTTCA

106 K  C  T  E  C  D  G  D  Y  A  E  K  Q  C  A
316 AAATGCACCGAGTGTGATGGAGACTATGCTGAAAAGCAATGCGCA

121 S  I  C  P  V  E  G  A  I  L  L  A  D  G  T
361 TCTATTTGTCCAGTTGAAGGTGCTATCTTGTTAGCAGACGGAACT

136 P  A  N  P  P  G  S  L  T  G  I  P  P  E  R
406 CCTGCTAACCCACCTGGTTCACTTACAGGAATCCCACCTGAAAGA

151 L  A  E  A  M  R  E  I  Q  A  R  *
451 TTGGCTGAGGCAATGAGAGAAATACAGGCAAGGTAA
```

SU9-**His10**-NifB*Av*

```
  1 M  A  S  T  R  V  L  A  S  R  L  A  S  Q  M
  1 ATGGCCTCCACTCGTGTCCTCGCCTCTCGCCTGGCCTCCCAGATG

 16 A  A  S  A  K  V  A  R  P  A  V  R  V  A  Q
 46 GCTGCTTCCGCCAAGGTTGCCCGCCCTGCTGTCCGCGTTGCTCAG

 31 V  S  K  R  T  I  Q  T  G  S  P  L  Q  T  L
 91 GTCAGCAAGCGCACCATCCAGACTGGCTCCCCCCTCCAGACCCTC

 46 K  R  T  Q  M  T  S  I  V  N  A  T  T  R  Q
136 AAGCGCACCCAGATGACCTCCATCGTCAACGCCACCACCCGCCAG

 61 A  F  Q  K  R  A  Y  S  S  M  H  H  H  H  H
181 GCTTTCCAGAAGCGCGCCTACTCTTCCATGCATCATCACCATCAC

 76 H  H  H  H  H  E  L  S  V  L  G  Q  N  N  G
226 CATCACCATCACCATGAATTGTCTGTTTTGGGTCAAAACAACGGT

 91 G  Q  H  S  A  G  G  C  S  S  S  S  C  G  S
271 GGTCAACACTCTGCTGGTGGTTGTTCTTCTTCTTCTTGTGGTTCT

106 T  H  D  Q  L  S  H  L  P  E  N  I  R  A  K
316 ACTCACGACCAATTGTCTCACTTGCCAGAAAACATCAGAGCTAAG

121 V  Q  N  H  P  C  Y  S  E  E  A  H  H  Y  F
361 GTTCAAAACCACCCATGTTACTCTGAAGAAGCTCACCACTACTTC
```

```
 136 A  R  M  H  V  A  V  A  P  A  C  N  I  Q  C
 406 GCTAGAATGCACGTTGCTGTTGCTCCAGCTTGTAACATCCAATGT

 151 H  Y  C  N  R  K  Y  D  C  A  N  E  S  R  P
 451 CACTACTGTAACAGAAAGTACGACTGTGCTAACGAATCTAGACCA

 166 G  V  V  S  E  V  L  T  P  E  Q  A  V  K  K
 496 GGTGTTGTTTCTGAAGTTTTGACTCCAGAACAAGCTGTTAAGAAG

 181 V  K  A  V  A  A  A  I  P  Q  M  S  V  L  G
 541 GTTAAGGCTGTTGCTGCTGCTATCCCACAAATGTCTGTTTTGGGT

 196 I  A  G  P  G  D  P  L  A  N  P  K  R  T  L
 586 ATCGCTGGTCCAGGTGACCCATTGGCTAACCCAAAGAGAACTTTG

 211 D  T  F  R  M  L  S  E  Q  A  P  D  I  K  L
 631 GACACTTTCAGAATGTTGTCTGAACAAGCTCCAGACATCAAGTTG

 226 C  V  S  T  N  G  L  A  L  P  E  C  V  E  E
 676 TGTGTTTCTACTAACGGTTTGGCTTTGCCAGAATGTGTTGAAGAA

 241 L  A  K  H  N  I  D  H  V  T  I  T  I  N  C
 721 TTGGCTAAGCACAACATCGACCACGTTACTATCACTATCAACTGT

 256 V  D  P  E  I  G  A  K  I  Y  P  W  I  Y  W
 766 GTTGACCCAGAAATCGGTGCTAAGATCTACCCATGGATCTACTGG

 271 N  N  K  R  I  R  G  V  K  A  A  K  I  L  I
 811 AACAACAAGAGAATCAGAGGTGTTAAGGCTGCTAAGATCTTGATC

 286 E  Q  Q  Q  K  G  L  E  M  L  V  A  R  G  I
 856 GAACAACAACAAAAGGGTTTGGAAATGTTGGTTGCTAGAGGTATC

 301 L  V  K  V  N  S  V  M  I  P  G  V  N  D  E
 901 TTGGTTAAGGTTAACTCTGTTATGATCCCAGGTGTTAACGACGAA

 316 H  L  K  E  V  S  K  I  V  K  A  K  G  A  F
 946 CACTTGAAGGAAGTTTCTAAGATCGTTAAGGCTAAGGGTGCTTTC

 331 L  H  N  V  M  P  L  I  A  E  P  E  H  G  T
 991 TTGCACAACGTTATGCCATTGATCGCTGAACCAGAACACGGTACT

 346 F  Y  G  V  M  G  Q  R  S  P  E  P  E  E  L
1036 TTCTACGGTGTTATGGGTCAAAGATCTCCAGAACCAGAAGAATTG

 361 Q  D  L  Q  D  A  C  A  G  D  M  N  M  M  R
1081 CAAGACTTGCAAGACGCTTGTGCTGGTGACATGAACATGATGAGA
```

376 H C R Q C R A D A V G M L G E
1126 CACTGTAGACAATGTAGAGCTGACGCTGTTGGTATGTTGGGTGAA

391 D R G D E F T L D K I E S M E
1171 GACAGAGGTGACGAATTCACTTTGGACAAGATCGAATCTATGGAA

406 I D Y E A A M V K R A A I H A
1216 ATCGACTACGAAGCTGCTATGGTTAAGAGAGCTGCTATCCACGCT

421 A I K E E L D E K A A K K E R
1261 GCTATCAAGGAAGAATTGGACGAAAAGGCTGCTAAGAAGGAAAGA

436 L A G L S V A S V Q N G T S G
1306 TTGGCTGGTTTGTCTGTTGCTTCTGTTCAAAACGGTACTTCTGGT

451 R Y R P V L M A V A T S G G G
1351 AGATACAGACCAGTTTTGATGGCTGTTGCTACTTCTGGTGGTGGT

466 L I N Q H F G H A T E F L V Y
1396 TTGATCAACCAACACTTCGGTCACGCTACTGAATTCTTGGTTTAC

481 E A S P S G V R F I G H R R V
1441 GAAGCTTCTCCATCTGGTGTTAGATTCATCGGTCACAGAAGAGTT

496 D Q Y C V G N D T C G E K E S
1486 GACCAATACTGTGTTGGTAACGACACTTGTGGTGAAAAGGAATCT

511 A L A G S I R A L K G C E A V
1531 GCTTTGGCTGGTTCTATCAGAGCTTTGAAGGGTTGTGAAGCTGTT

526 L C S K I G F E P W S D L E T
1576 TTGTGTTCTAAGATCGGTTTCGAACCATGGTCTGACTTGGAAACT

541 A G I Q P N G E H A M E P I E
1621 GCTGGTATCCAACCAAACGGTGAACACGCTATGGAACCAATCGAA

556 E A V M A V Y R E M I E S G R
1666 GAAGCTGTTATGGCTGTTTACAGAGAAATGATCGAATCTGGTAGA

571 L E N D G A L L Q A K A *
1711 TTGGAAAACGACGGTGCTTTGTTGCAAGCTAAGGCTTAA

SU9-**NifB**$_{Av}$-His$_{10}$

1 M A S T R V L A S R L A S Q M
1 ATGGCCTCCACTCGTGTCCTCGCCTCTCGCCTGGCCTCCCAGATG

16 A A S A K V A R P A V R V A Q
46 GCTGCTTCCGCCAAGGTTGCCCGCCCTGCTGTCCGCGTTGCTCAG

31 V S K R T I Q T G S P L Q T L
91 GTCAGCAAGCGCACCATCCAGACTGGCTCCCCCCTCCAGACCCTC

46 K R T Q M T S I V N A T T R Q
136 AAGCGCACCCAGATGACCTCCATCGTCAACGCCACCACCCGCCAG

61 A F Q K R A Y S S **M E L S V L**
181 GCTTTCCAGAAGCGCGCCTACTCTTCCATGGAATTGTCTGTTTTG

76 **G Q N N G G Q H S A G G C S S**
226 GGTCAAAACAACGGTGGTCAACACTCTGCTGGTGGTTGTTCTTCT

91 **S S C G S T H D Q L S H L P E**
271 TCTTCTTGTGGTTCTACTCACGACCAATTGTCTCACTTGCCAGAA

106 **N I R A K V Q N H P C Y S E E**
316 AACATCAGAGCTAAGGTTCAAAACCACCCATGTTACTCTGAAGAA

121 **A H H Y F A R M H V A V A P A**
361 GCTCACCACTACTTCGCTAGAATGCACGTTGCTGTTGCTCCAGCT

136 **C N I Q C H Y C N R K Y D C A**
406 TGTAACATCCAATGTCACTACTGTAACAGAAAGTACGACTGTGCT

151 **N E S R P G V V S E V L T P E**
451 AACGAATCTAGACCAGGTGTTGTTTCTGAAGTTTTGACTCCAGAA

166 **Q A V K K V K A V A A A I P Q**
496 CAAGCTGTTAAGAAGGTTAAGGCTGTTGCTGCTGCTATCCCACAA

181 **M S V L G I A G P G D P L A N**
541 ATGTCTGTTTTGGGTATCGCTGGTCCAGGTGACCCATTGGCTAAC

196 **P K R T L D T F R M L S E Q A**
586 CCAAAGAGAACTTTGGACACTTTCAGAATGTTGTCTGAACAAGCT

211 **P D I K L C V S T N G L A L P**
631 CCAGACATCAAGTTGTGTGTTTCTACTAACGGTTTGGCTTTGCCA

226 **E C V E E L A K H N I D H V T**
676 GAATGTGTTGAAGAATTGGCTAAGCACAACATCGACCACGTTACT

241 **I T I N C V D P E I G A K I Y**
721 ATCACTATCAACTGTGTTGACCCAGAAATCGGTGCTAAGATCTAC

256 **P W I Y W N N K R I R G V K A**
766 CCATGGATCTACTGGAACAACAAGAGAATCAGAGGTGTTAAGGCT

271 **A K I L I E Q Q Q K G L E M L**
811 GCTAAGATCTTGATCGAACAACAACAAAAGGGTTTGGAAATGTTG

286 **V A R G I L V K V N S V M I P**
856 GTTGCTAGAGGTATCTTGGTTAAGGTTAACTCTGTTATGATCCCA

301 **G V N D E H L K E V S K I V K**
901 GGTGTTAACGACGAACACTTGAAGGAAGTTTCTAAGATCGTTAAG

316 **A K G A F L H N V M P L I A E**
946 GCTAAGGGTGCTTTCTTGCACAACGTTATGCCATTGATCGCTGAA

331 **P E H G T F Y G V M G Q R S P**
991 CCAGAACACGGTACTTTCTACGGTGTTATGGGTCAAAGATCTCCA

346 **E P E E L Q D L Q D A C A G D**
1036 GAACCAGAAGAATTGCAAGACTTGCAAGACGCTTGTGCTGGTGAC

361 **M N M M R H C R Q C R A D A V**
1081 ATGAACATGATGAGACACTGTAGACAATGTAGAGCTGACGCTGTT

376 **G M L G E D R G D E F T L D K**
1126 GGTATGTTGGGTGAAGACAGAGGTGACGAATTCACTTTGGACAAG

391 **I E S M E I D Y E A A M V K R**
1171 ATCGAATCTATGGAAATCGACTACGAAGCTGCTATGGTTAAGAGA

406 **A A I H A A I K E E L D E K A**
1216 GCTGCTATCCACGCTGCTATCAAGGAAGAATTGGACGAAAAGGCT

421 **A K K E R L A G L S V A S V Q**
1261 GCTAAGAAGGAAAGATTGGCTGGTTTGTCTGTTGCTTCTGTTCAA

436 **N G T S G R Y R P V L M A V A**
1306 AACGGTACTTCTGGTAGATACAGACCAGTTTTGATGGCTGTTGCT

451 **T S G G G L I N Q H F G H A T**
1351 ACTTCTGGTGGTGGTTTGATCAACCAACACTTCGGTCACGCTACT

466 **E F L V Y E A S P S G V R F I**
1396 GAATTCTTGGTTTACGAAGCTTCTCCATCTGGTGTTAGATTCATC

481 **G H R R V D Q Y C V G N D T C**
1441 GGTCACAGAAGAGTTGACCAATACTGTGTTGGTAACGACACTTGT

496 **G E K E S A L A G S I R A L K**
1486 GGTGAAAAGGAATCTGCTTTGGCTGGTTCTATCAGAGCTTTGAAG

511 **G C E A V L C S K I G F E P W**
1531 GGTTGTGAAGCTGTTTTGTGTTCTAAGATCGGTTTCGAACCATGG

526 **S D L E T A G I Q P N G E H A**
1576 TCTGACTTGGAAACTGCTGGTATCCAACCAAACGGTGAACACGCT

541 **M E P I E E A V M A V Y R E M**
1621 ATGGAACCAATCGAAGAAGCTGTTATGGCTGTTTACAGAGAAATG

556 **I E S G R L E N D G A L L Q A**
1666 ATCGAATCTGGTAGATTGGAAAACGACGGTGCTTTGTTGCAAGCT

571 **K A** H H H H H H H H H H *
1711 AAGGCTCATCATCATCACCACCACCATCATCACCATTAA

SU9-**NifB$_{Mi}$**-His$_{10}$

1 M A S T R V L A S R L A S Q M
1 ATGGCCTCCACTCGTGTCCTCGCCTCTCGCCTGGCCTCCCAGATG

16 A A S A K V A R P A V R V A Q
46 GCTGCTTCCGCCAAGGTTGCCCGCCCTGCTGTCCGCGTTGCTCAG

31 V S K R T I Q T G S P L Q T L
91 GTCAGCAAGCGCACCATCCAGACTGGCTCCCCCCTCCAGACCCTC

46 K R T Q M T S I V N A T T R Q
136 AAGCGCACCCAGATGACCTCCATCGTCAACGCCACCACCCGCCAG

61 A F Q K R A Y S S **M E K M S K**
181 GCTTTCCAGAAGCGCGCCTACTCTTCCATGGAGAAAATGTCTAAA

76 **F S H L L K A H P C F N E K V**
226 TTTTCTCATTTGCTTAAGGCTCACCCATGTTTCAACGAAAAGGTT

91 **H D K Y G R V H L P V A P R C**
271 CATGATAAGTATGGTAGAGTTCACTTGCCAGTGGCTCCTAGGTGC

106 **N I A C K F C K R S V S K E C**
316 AACATCGCATGTAAATTTTGCAAGAGATCTGTTTCAAAGGAATGT

121 **C E H R P G V S L G V L K P E**
361 TGCGAGCATAGGCCAGGTGTTAGTTTGGGAGTGTTAAAGCCTGAA

136 **D V E D Y L K K I L K E M P N**
406 GATGTGGAGGACTATTTGAAGAAAATTCTTAAGGAAATGCCAAAT

151 **I K V V G I A G P G D S L F N**
451 ATTAAGGTTGTGGGTATCGCTGGTCCTGGAGATTCCTTGTTTAAT

166 **K E T F E T L K I I D E K F P**
496 AAGGAGACTTTCGAGACACTTAAAATTATCGACGAAAAGTTCCCA

181 **N L I K C I S T N G L L L S K**
541 AATCTTATTAAGTGTATCTCTACAAACGGACTTTTGCTTTCAAAA

196 **Y Y K D L A N L N V R T I T V**
586 TATTACAAGGATCTTGCTAATTTGAACGTTAGAACTATCACAGTT

211 **T V N A I K P E I L E K I V D**
631 ACCGTGAATGCAATTAAACCTGAAATCTTGGAGAAGATAGTTGAT

226 **W V Y Y D K K L Y R G L E G A**
676 TGGGTGTACTACGACAAAAAGTTGTACAGAGGTCTTGAAGGAGCT

241 **K L L I E K Q I E G I K K A S**
721 AAGCTTTTGATCGAAAAGCAAATCGAGGGAATTAAAAAGGCTAGT

256 **E E D F I I K I N T V L I P E**
766 GAAGAGGATTTCATAATTAAAATCAATACCGTTCTTATCCCTGAG

271 **I N M D H V V E I A K F F K D**
811 ATAAACATGGATCACGTTGTGGAAATCGCTAAGTTTTTCAAGGAC

286 **Y A Y V Q N I I P L I P Q Y K**
856 TACGCATACGTTCAAAACATCATACCATTGATCCCTCAGTATAAG

301 **M K E L R A P T C E E I K K V**
901 ATGAAAGAACTTAGAGCTCCAACTTGTGAAGAAATTAAGAAAGTT

316 **R K E C E K Y I P Q F R A C G**
946 AGAAAGGAATGCGAGAAGTACATTCCTCAATTCAGAGCATGTGGA

331 **Q C R A D A V G L I K E K E L**
991 CAGTGCAGGGCTGATGCAGTGGGATTGATTAAAGAAAAGGAGCTT

346 **L K E F F K E K N K E K N I K**
1036 CTTAAGGAATTTTTCAAGGAAAAGAATAAGGAAAAGAACATCAAA

361 **L E V F D L K H F S H** H H H H
1081 CTTGAAGTTTTCGACTTGAAGCATTTCTCACACCATCATCATCAC

```
 376 H  H  H  H  H  H  *
1126 CACCACCATCATCACCATTAA
```

SU9-**FdxN**-HA

```
   1 M  A  S  T  R  V  L  A  S  R  L  A  S  Q  M
   1 ATGGCCTCCACTCGTGTCCTCGCCTCTCGCCTGGCCTCCCAGATG

  16 A  A  S  A  K  V  A  R  P  A  V  R  V  A  Q
  46 GCTGCTTCCGCCAAGGTTGCCCGCCCTGCTGTCCGCGTTGCTCAG

  31 V  S  K  R  T  I  Q  T  G  S  P  L  Q  T  L
  91 GTCAGCAAGCGCACCATCCAGACTGGCTCCCCCCTCCAGACCCTC

  46 K  R  T  Q  M  T  S  I  V  N  A  T  T  R  Q
 136 AAGCGCACCCAGATGACCTCCATCGTCAACGCCACCACCCGCCAG

  61 A  F  Q  K  R  A  Y  S  S  M  A  L  K  I  V
 181 GCTTTCCAGAAGCGCGCCTACTCTTCCATGGCTCTTAAGATAGTT

  76 E  S  C  V  N  C  W  A  C  V  D  V  C  P  S
 226 GAGTCTTGTGTGAACTGCTGGGCATGTGTTGATGTGTGCCCAAGT

  91 E  A  I  S  L  A  G  P  H  F  E  I  S  A  S
 271 GAGGCTATATCCTTGGCAGGTCCTCATTTTGAAATTTCTGCTTCA

 106 K  C  T  E  C  D  G  D  Y  A  E  K  Q  C  A
 316 AAATGCACCGAGTGTGATGGAGACTATGCTGAAAAGCAATGCGCA

 121 S  I  C  P  V  E  G  A  I  L  L  A  D  G  T
 361 TCTATTTGTCCAGTTGAAGGTGCTATCTTGTTAGCAGACGGAACT

 136 P  A  N  P  P  G  S  L  T  G  I  P  P  E  R
 406 CCTGCTAACCCACCTGGTTCACTTACAGGAATCCCACCTGAAAGA

 151 L  A  E  A  M  R  E  I  Q  A  R  Y  P  Y  D
 451 TTGGCTGAGGCAATGAGAGAAATACAGGCAAGGTATCCATATGAT

 166 V  P  D  Y  A  *
 496 GTTCCAGATTATGCTTAA
```

SUPPLEMENTARY FIGURE 2. Codon-optimized DNA sequences and translated protein sequences. Sequences of the *nifU*, *nifS*, *fdxN*, and *nifB* genes and their corresponding proteins expressed from yeast expression vectors shown in Supplementary Figure 1. Amino acids highlighted in green were identified by N-terminal sequencing.

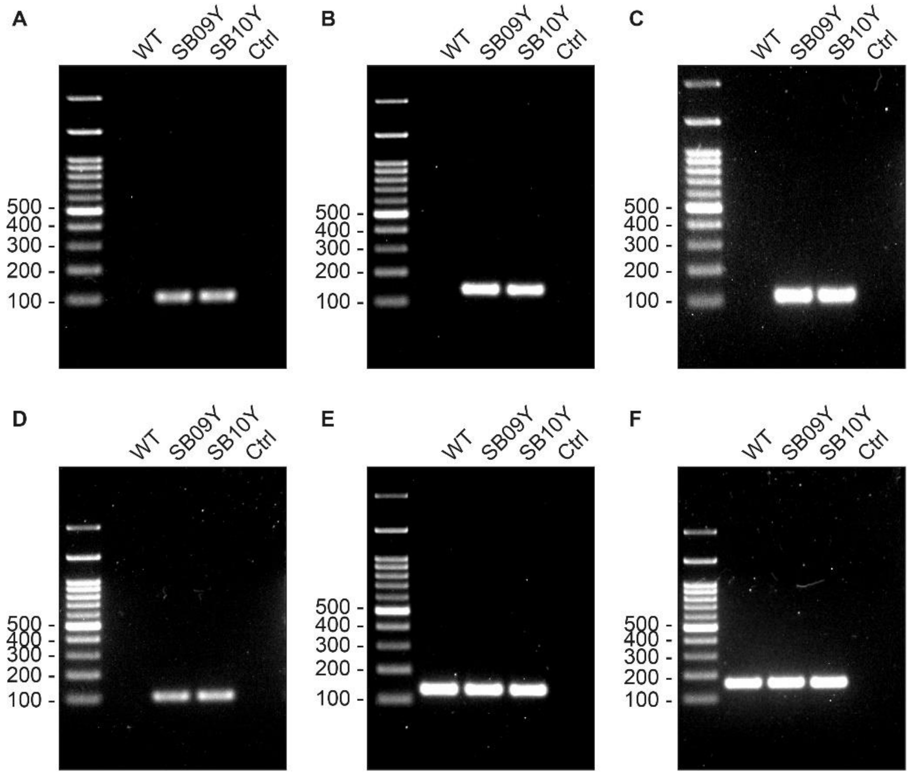

G

	WT	SB09Y	SB10Y	Ctrl
fdxN (A)	nd	17.1	16.3	nd
fdxN (C)	nd	15.1	14.7	nd
rdn18 (E)	8.4	8.0	7.3	nd
alg9 (F)	22.3	20.7	20.3	nd

SUPPLEMENTARY FIGURE 3. Detection of *fdxN* transcripts in yeast strains SB09Y and SB10Y. (A-F) Reverse transcription polymerase chain reaction (RT-PCR) to detect *fdxN* transcripts in SB09Y and SB10Y using 4 distinct primer combinations. Primer sequences are detailed in Material and Methods. Expected amplicon sizes were: A, 105 bp; B, 144 bp; C, 110 bp, and D, 105 bp, respectively. Primers amplifying transcripts of *rdn18* (E, 133 bp) and *alg9* (F, 162 bp) were used as cDNA positive controls. PCR in absence of cDNA (Ctrl) was used as negative control in all cases. **(G)** Ct values obtained by real-time polymerase chain reaction (qPCR) using two primer combinations for *fdxN*, in addition to single primer combinations for *rdn18* and *alg9*. The letter within brackets indicate the RT-PCR primer combination used. Nd means not detected within 40 cycles of amplification.

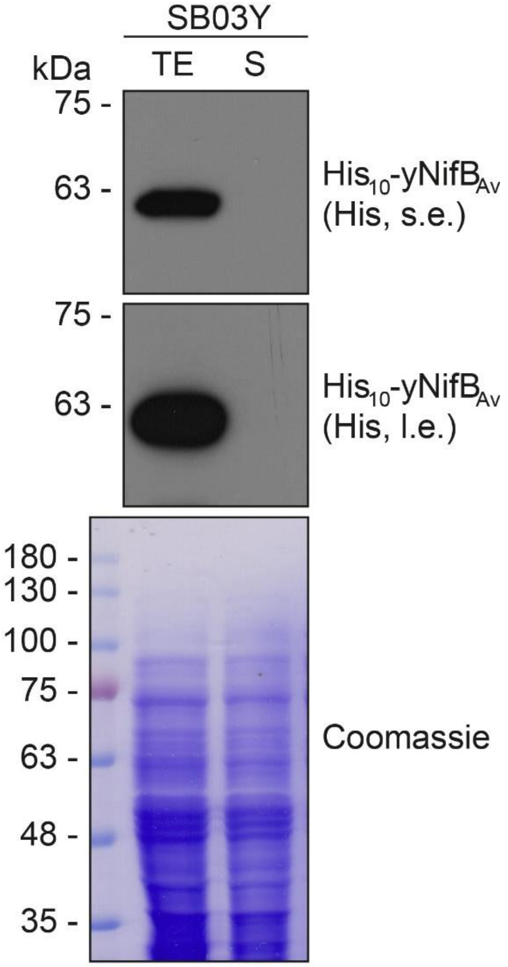

SUPPLEMENTARY FIGURE 4. Solubility of N-terminally His-tagged NifB*Av* expressed in *S. cerevisiae*. Western blot analysis of mitochondria targeted His_{10}-NifB*Av* in total protein extracts (TE) and the soluble fraction (S) from SB03Y. Short (s.e.) and long (l.e.) exposures of the same membranes are shown. Coomassie stained SDS-PAGE (below) of the protein extracts is included as loading control.

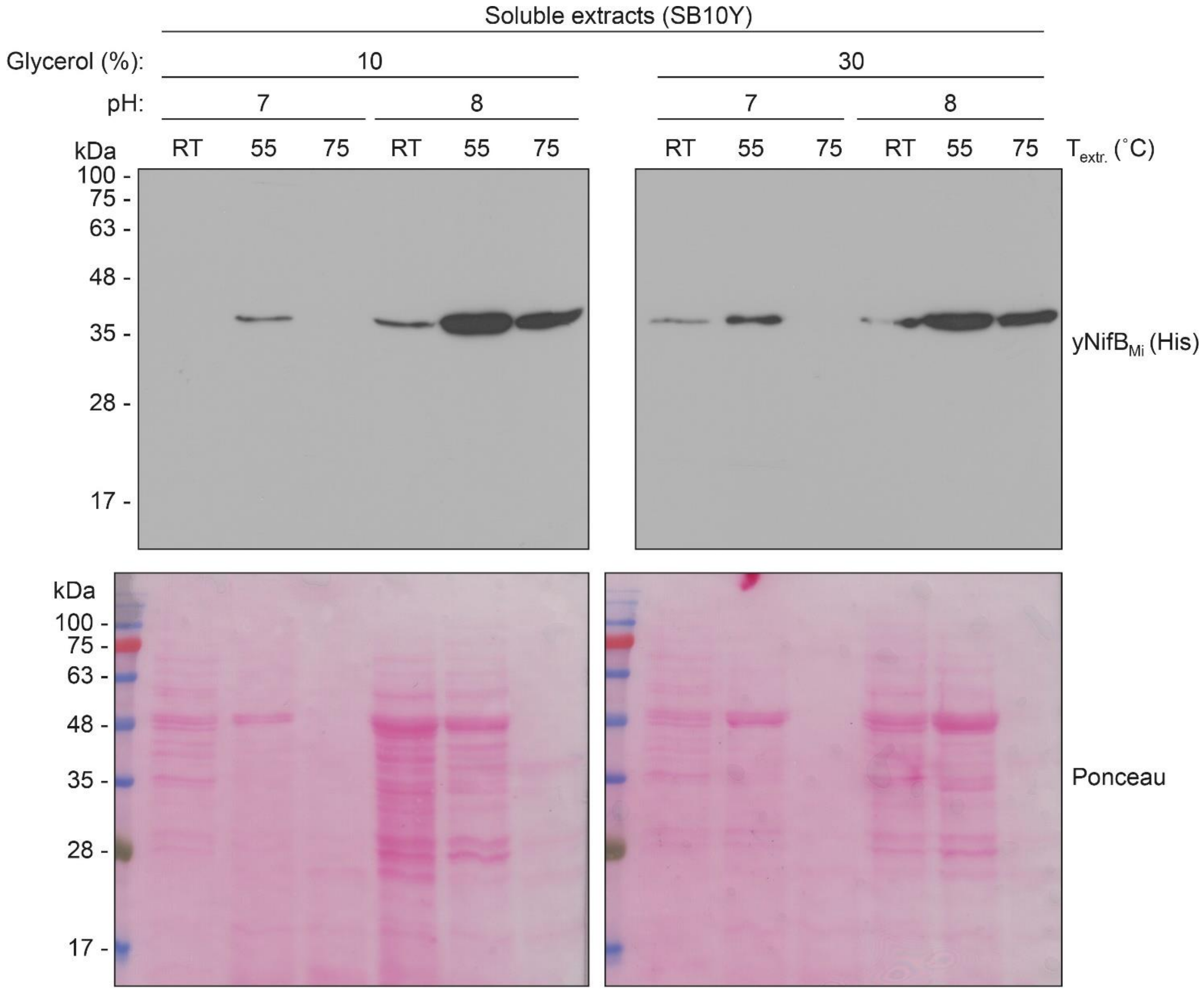

SUPPLEMENTARY FIGURE 5. Levels of soluble yNifB*Mi* in SB10Y obtained by using 12 different extraction conditions. Protein extracts were resolved by SDS-PAGE and then transferred to membranes for immunoblot analysis. Western blot membranes were exposed together on the same film. Ponceau stainings of the same membranes show the levels of total yeast proteins in each extracted sample.

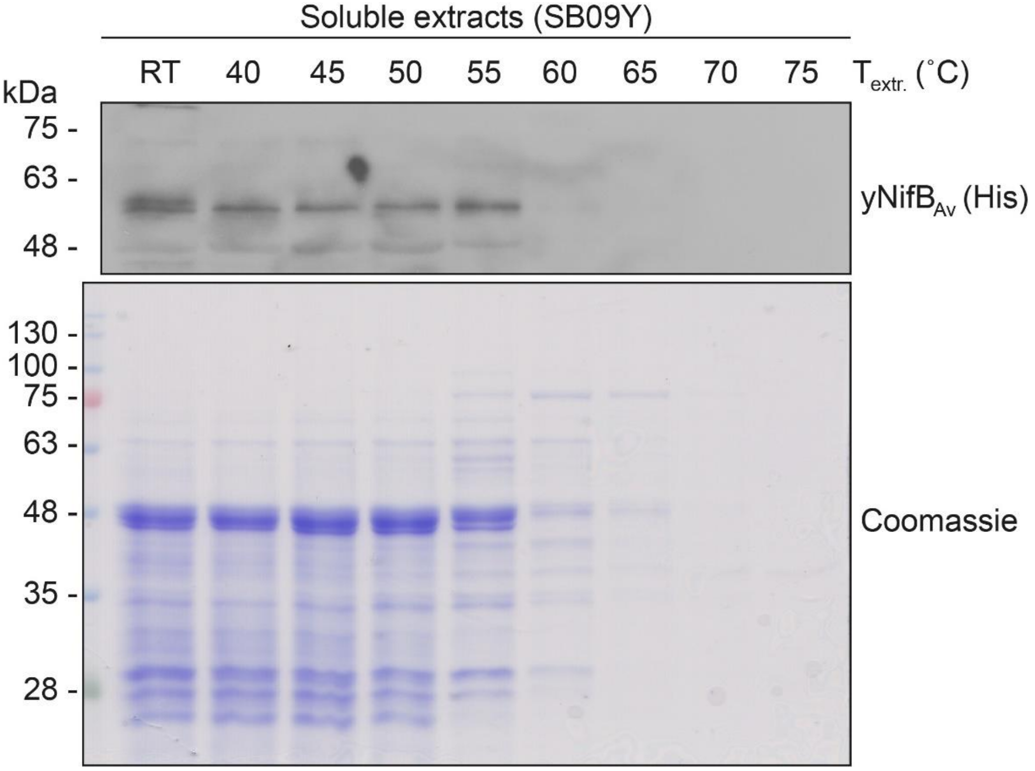

SUPPLEMENTARY FIGURE 6. Levels of soluble yNifB$_{Av}$ upon heat-treatment of SB09Y extracts. SDS-PAGE and Western blot analysis showing levels of soluble yNifB$_{Av}$ in SB09Y protein extracts upon heat-treatment at increasing temperatures. Heat-precipitation of yeast proteins at the different temperatures is shown using Coomassie stained SDS gels loaded with the treated extracts.

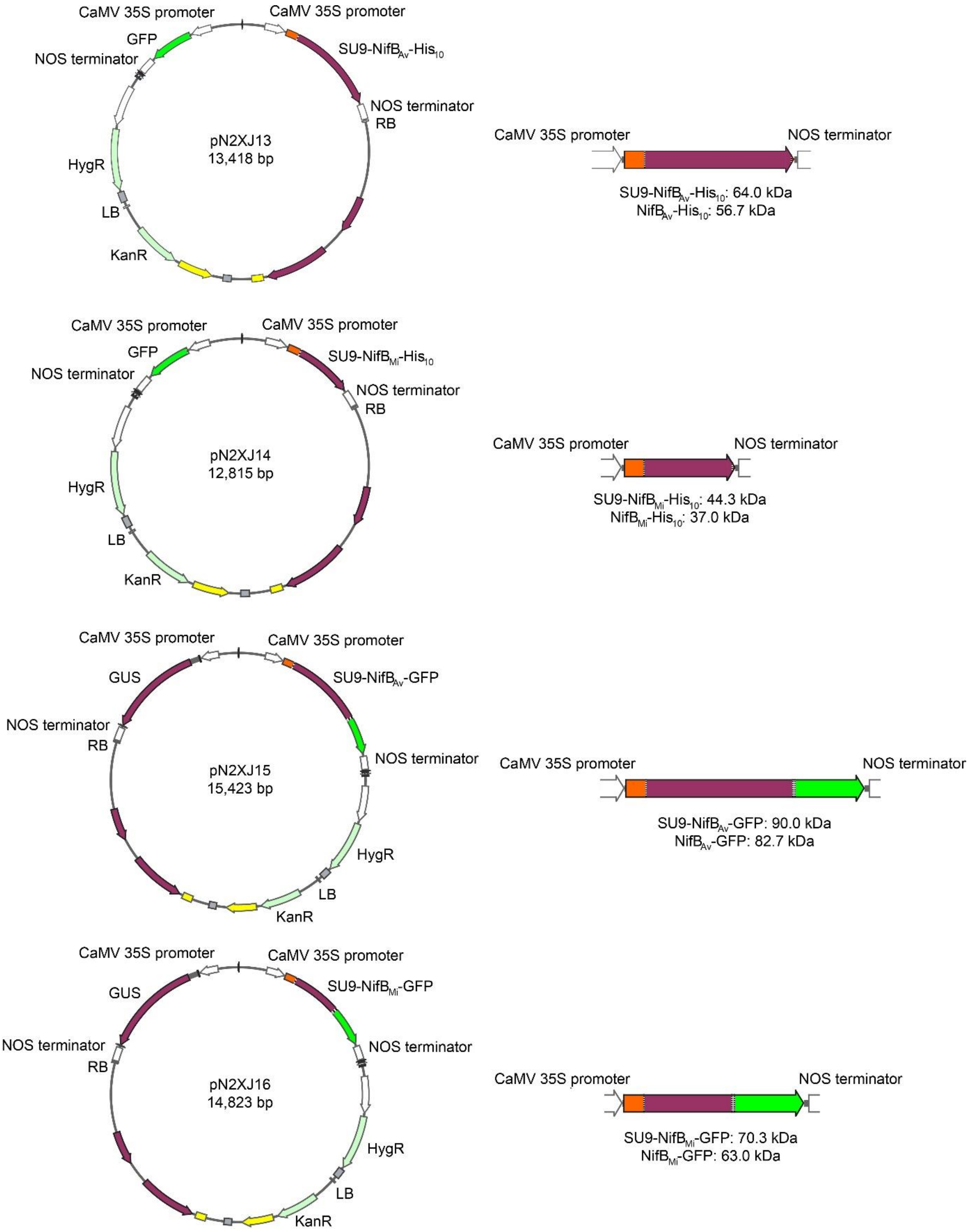

SUPPLEMENTARY FIGURE 7. Plant expression vectors with SU9 leader sequence. Schematic overview of plant expression vectors for expression of SU9-NifB$_{Av}$-His$_{10}$, SU9-NifB$_{Mi}$-His$_{10}$, SU9-NifB$_{Av}$-GFP and SU9-NifB$_{Mi}$-GFP. See Supplementary Figure 8 for detailed information about DNA and protein sequences.

SU9-**NifB**$_{Av}$-His$_{10}$

```
  1 M  V  D  P  M  A  S  T  R  V  L  A  S  R  L
  1 ATGGTAGATCCAATGGCCTCCACTCGTGTCCTCGCCTCTCGCCTG

 16 A  S  Q  M  A  A  S  A  K  V  A  R  P  A  V
 46 GCCTCCCAGATGGCTGCTTCCGCCAAGGTTGCCCGCCCTGCTGTC

 31 R  V  A  Q  V  S  K  R  T  I  Q  T  G  S  P
 91 CGCGTTGCTCAGGTCAGCAAGCGCACCATCCAGACTGGCTCCCCC

 46 L  Q  T  L  K  R  T  Q  M  T  S  I  V  N  A
136 CTCCAGACCCTCAAGCGCACCCAGATGACCTCCATCGTCAACGCC

 61 T  T  R  Q  A  F  Q  K  R  A  Y  S  S  M  E
181 ACCACCCGCCAGGCTTTCCAGAAGCGCGCCTACTCTTCCATGGAA

 76 L  S  V  L  G  Q  N  N  G  G  Q  H  S  A  G
226 TTGTCTGTTTTGGGTCAAAACAACGGTGGTCAACACTCTGCTGGT

 91 G  C  S  S  S  S  C  G  S  T  H  D  Q  L  S
271 GGTTGTTCTTCTTCTTCTTGTGGTTCTACTCACGACCAATTGTCT

106 H  L  P  E  N  I  R  A  K  V  Q  N  H  P  C
316 CACTTGCCAGAAAACATCAGAGCTAAGGTTCAAAACCACCCATGT

121 Y  S  E  E  A  H  H  Y  F  A  R  M  H  V  A
361 TACTCTGAAGAAGCTCACCACTACTTCGCTAGAATGCACGTTGCT

136 V  A  P  A  C  N  I  Q  C  H  Y  C  N  R  K
406 GTTGCTCCAGCTTGTAACATCCAATGTCACTACTGTAACAGAAAG

151 Y  D  C  A  N  E  S  R  P  G  V  V  S  E  V
451 TACGACTGTGCTAACGAATCTAGACCAGGTGTTGTTTCTGAAGTT

166 L  T  P  E  Q  A  V  K  K  V  K  A  V  A  A
496 TTGACTCCAGAACAAGCTGTTAAGAAGGTTAAGGCTGTTGCTGCT

181 A  I  P  Q  M  S  V  L  G  I  A  G  P  G  D
541 GCTATCCCACAAATGTCTGTTTTGGGTATCGCTGGTCCAGGTGAC

196 P  L  A  N  P  K  R  T  L  D  T  F  R  M  L
586 CCATTGGCTAACCCAAAGAGAACTTTGGACACTTTCAGAATGTTG

211 S  E  Q  A  P  D  I  K  L  C  V  S  T  N  G
631 TCTGAACAAGCTCCAGACATCAAGTTGTGTGTTTCTACTAACGGT

226 L  A  L  P  E  C  V  E  E  L  A  K  H  N  I
676 TTGGCTTTGCCAGAATGTGTTGAAGAATTGGCTAAGCACAACATC
```

241 **D H V T I T I N C V D P E I G**
721 GACCACGTTACTATCACTATCAACTGTGTTGACCCAGAAATCGGT

256 **A K I Y P W I Y W N N K R I R**
766 GCTAAGATCTACCCATGGATCTACTGGAACAACAAGAGAATCAGA

271 **G V K A A K I L I E Q Q Q K G**
811 GGTGTTAAGGCTGCTAAGATCTTGATCGAACAACAACAAAAGGGT

286 **L E M L V A R G I L V K V N S**
856 TTGGAAATGTTGGTTGCTAGAGGTATCTTGGTTAAGGTTAACTCT

301 **V M I P G V N D E H L K E V S**
901 GTTATGATCCCAGGTGTTAACGACGAACACTTGAAGGAAGTTTCT

316 **K I V K A K G A F L H N V M P**
946 AAGATCGTTAAGGCTAAGGGTGCTTTCTTGCACAACGTTATGCCA

331 **L I A E P E H G T F Y G V M G**
991 TTGATCGCTGAACCAGAACACGGTACTTTCTACGGTGTTATGGGT

346 **Q R S P E P E E L Q D L Q D A**
1036 CAAAGATCTCCAGAACCAGAAGAATTGCAAGACTTGCAAGACGCT

361 **C A G D M N M M R H C R Q C R**
1081 TGTGCTGGTGACATGAACATGATGAGACACTGTAGACAATGTAGA

376 **A D A V G M L G E D R G D E F**
1126 GCTGACGCTGTTGGTATGTTGGGTGAAGACAGAGGTGACGAATTC

391 **T L D K I E S M E I D Y E A A**
1171 ACTTTGGACAAGATCGAATCTATGGAAATCGACTACGAAGCTGCT

406 **M V K R A A I H A A I K E E L**
1216 ATGGTTAAGAGAGCTGCTATCCACGCTGCTATCAAGGAAGAATTG

421 **D E K A A K K E R L A G L S V**
1261 GACGAAAAGGCTGCTAAGAAGGAAAGATTGGCTGGTTTGTCTGTT

436 **A S V Q N G T S G R Y R P V L**
1306 GCTTCTGTTCAAAACGGTACTTCTGGTAGATACAGACCAGTTTTG

451 **M A V A T S G G G L I N Q H F**
1351 ATGGCTGTTGCTACTTCTGGTGGTGGTTTGATCAACCAACACTTC

466 **G H A T E F L V Y E A S P S G**
1396 GGTCACGCTACTGAATTCTTGGTTTACGAAGCTTCTCCATCTGGT

481 **V R F I G H R R V D Q Y C V G**
1441 GTTAGATTCATCGGTCACAGAAGAGTTGACCAATACTGTGTTGGT

496 **N D T C G E K E S A L A G S I**
1486 AACGACACTTGTGGTGAAAAGGAATCTGCTTTGGCTGGTTCTATC

511 **R A L K G C E A V L C S K I G**
1531 AGAGCTTTGAAGGGTTGTGAAGCTGTTTTGTGTTCTAAGATCGGT

526 **F E P W S D L E T A G I Q P N**
1576 TTCGAACCATGGTCTGACTTGGAAACTGCTGGTATCCAACCAAAC

541 **G E H A M E P I E E A V M A V**
1621 GGTGAACACGCTATGGAACCAATCGAAGAAGCTGTTATGGCTGTT

556 **Y R E M I E S G R L E N D G A**
1666 TACAGAGAAATGATCGAATCTGGTAGATTGGAAAACGACGGTGCT

571 **L L Q A K A** H H H H H H H H H
1711 TTGTTGCAAGCTAAGGCTCATCATCATCACCACCACCATCATCAC

586 H *
1756 CATTAA

SU9-**NifB**$_{Mi}$-His$_{10}$
1 M V D P M A S T R V L A S R L
1 ATGGTAGATCCAATGGCCTCCACTCGTGTCCTCGCCTCTCGCCTG

16 A S Q M A A S A K V A R P A V
46 GCCTCCCAGATGGCTGCTTCCGCCAAGGTTGCCCGCCCTGCTGTC

31 R V A Q V S K R T I Q T G S P
91 CGCGTTGCTCAGGTCAGCAAGCGCACCATCCAGACTGGCTCCCCC

46 L Q T L K R T Q M T S I V N A
136 CTCCAGACCCTCAAGCGCACCCAGATGACCTCCATCGTCAACGCC

61 T T R Q A F Q K R A Y S S **M E**
181 ACCACCCGCCAGGCTTTCCAGAAGCGCGCCTACTCTTCCATGGAG

76 **K M S K F S H L L K A H P C F**
226 AAAATGTCTAAATTTTCTCATTTGCTTAAGGCTCACCCATGTTTC

91 **N E K V H D K Y G R V H L P V**
271 AACGAAAAGGTTCATGATAAGTATGGTAGAGTTCACTTGCCAGTG

106 **A P R C N I A C K F C K R S V**
316 GCTCCTAGGTGCAACATCGCATGTAAATTTTGCAAGAGATCTGTT

121 **S K E C C E H R P G V S L G V**
361 TCAAAGGAATGTTGCGAGCATAGGCCAGGTGTTAGTTTGGGAGTG

136 **L K P E D V E D Y L K K I L K**
406 TTAAAGCCTGAAGATGTGGAGGACTATTTGAAGAAAATTCTTAAG

151 **E M P N I K V V G I A G P G D**
451 GAAATGCCAAATATTAAGGTTGTGGGTATCGCTGGTCCTGGAGAT

166 **S L F N K E T F E T L K I I D**
496 TCCTTGTTTAATAAGGAGACTTTCGAGACACTTAAAATTATCGAC

181 **E K F P N L I K C I S T N G L**
541 GAAAAGTTCCCAAATCTTATTAAGTGTATCTCTACAAACGGACTT

196 **L L S K Y Y K D L A N L N V R**
586 TTGCTTTCAAAATATTACAAGGATCTTGCTAATTTGAACGTTAGA

211 **T I T V T V N A I K P E I L E**
631 ACTATCACAGTTACCGTGAATGCAATTAAACCTGAAATCTTGGAG

226 **K I V D W V Y Y D K K L Y R G**
676 AAGATAGTTGATTGGGTGTACTACGACAAAAAGTTGTACAGAGGT

241 **L E G A K L L I E K Q I E G I**
721 CTTGAAGGAGCTAAGCTTTTGATCGAAAAGCAAATCGAGGGAATT

256 **K K A S E E D F I I K I N T V**
766 AAAAAGGCTAGTGAAGAGGATTTCATAATTAAAATCAATACCGTT

271 **L I P E I N M D H V V E I A K**
811 CTTATCCCTGAGATAAACATGGATCACGTTGTGGAAATCGCTAAG

286 **F F K D Y A Y V Q N I I P L I**
856 TTTTTCAAGGACTACGCATACGTTCAAAACATCATACCATTGATC

301 **P Q Y K M K E L R A P T C E E**
901 CCTCAGTATAAGATGAAAGAACTTAGAGCTCCAACTTGTGAAGAA

316 **I K K V R K E C E K Y I P Q F**
946 ATTAAGAAAGTTAGAAAGGAATGCGAGAAGTACATTCCTCAATTC

331 **R A C G Q C R A D A V G L I K**
991 AGAGCATGTGGACAGTGCAGGGCTGATGCAGTGGGATTGATTAAA

346 **E K E L L K E F F K E K N K E**
1036 GAAAAGGAGCTTCTTAAGGAATTTTTCAAGGAAAAGAATAAGGAA

361 **K N I K L E V F D L K H F S H**
1081 AAGAACATCAAACTTGAAGTTTTCGACTTGAAGCATTTCTCACAC

376 H H H H H H H H H H *
1126 CATCATCATCACCACCACCATCATCACCATTAA

SU9-**NifB*Av***-GFP

1 M A S T R V L A S R L A S Q M
1 ATGGCCTCCACTCGTGTCCTCGCCTCTCGCCTGGCCTCCCAGATG

16 A A S A K V A R P A V R V A Q
46 GCTGCTTCCGCCAAGGTTGCCCGCCCTGCTGTCCGCGTTGCTCAG

31 V S K R T I Q T G S P L Q T L
91 GTCAGCAAGCGCACCATCCAGACTGGCTCCCCCCTCCAGACCCTC

46 K R T Q M T S I V N A T T R Q
136 AAGCGCACCCAGATGACCTCCATCGTCAACGCCACCACCCGCCAG

61 A F Q K R A Y S S **M E L S V L**
181 GCTTTCCAGAAGCGCGCCTACTCTTCCATGGAATTGTCTGTTTTG

76 **G Q N N G G Q H S A G G C S S**
226 GGTCAAAACAACGGTGGTCAACACTCTGCTGGTGGTTGTTCTTCT

91 **S S C G S T H D Q L S H L P E**
271 TCTTCTTGTGGTTCTACTCACGACCAATTGTCTCACTTGCCAGAA

106 **N I R A K V Q N H P C Y S E E**
316 AACATCAGAGCTAAGGTTCAAAACCACCCATGTTACTCTGAAGAA

121 **A H H Y F A R M H V A V A P A**
361 GCTCACCACTACTTCGCTAGAATGCACGTTGCTGTTGCTCCAGCT

136 **C N I Q C H Y C N R K Y D C A**
406 TGTAACATCCAATGTCACTACTGTAACAGAAAGTACGACTGTGCT

151 **N E S R P G V V S E V L T P E**
451 AACGAATCTAGACCAGGTGTTGTTTCTGAAGTTTTGACTCCAGAA

166 **Q A V K K V K A V A A A I P Q**
496 CAAGCTGTTAAGAAGGTTAAGGCTGTTGCTGCTGCTATCCCACAA

181 **M S V L G I A G P G D P L A N**
541 ATGTCTGTTTTGGGTATCGCTGGTCCAGGTGACCCATTGGCTAAC

196 **P K R T L D T F R M L S E Q A**
586 CCAAAGAGAACTTTGGACACTTTCAGAATGTTGTCTGAACAAGCT

211 **P D I K L C V S T N G L A L P**
631 CCAGACATCAAGTTGTGTGTTTCTACTAACGGTTTGGCTTTGCCA

226 **E C V E E L A K H N I D H V T**
676 GAATGTGTTGAAGAATTGGCTAAGCACAACATCGACCACGTTACT

241 **I T I N C V D P E I G A K I Y**
721 ATCACTATCAACTGTGTTGACCCAGAAATCGGTGCTAAGATCTAC

256 **P W I Y W N N K R I R G V K A**
766 CCATGGATCTACTGGAACAACAAGAGAATCAGAGGTGTTAAGGCT

271 **A K I L I E Q Q Q K G L E M L**
811 GCTAAGATCTTGATCGAACAACAACAAAAGGGTTTGGAAATGTTG

286 **V A R G I L V K V N S V M I P**
856 GTTGCTAGAGGTATCTTGGTTAAGGTTAACTCTGTTATGATCCCA

301 **G V N D E H L K E V S K I V K**
901 GGTGTTAACGACGAACACTTGAAGGAAGTTTCTAAGATCGTTAAG

316 **A K G A F L H N V M P L I A E**
946 GCTAAGGGTGCTTTCTTGCACAACGTTATGCCATTGATCGCTGAA

331 **P E H G T F Y G V M G Q R S P**
991 CCAGAACACGGTACTTTCTACGGTGTTATGGGTCAAAGATCTCCA

346 **E P E E L Q D L Q D A C A G D**
1036 GAACCAGAAGAATTGCAAGACTTGCAAGACGCTTGTGCTGGTGAC

361 **M N M M R H C R Q C R A D A V**
1081 ATGAACATGATGAGACACTGTAGACAATGTAGAGCTGACGCTGTT

376 **G M L G E D R G D E F T L D K**
1126 GGTATGTTGGGTGAAGACAGAGGTGACGAATTCACTTTGGACAAG

391 **I E S M E I D Y E A A M V K R**
1171 ATCGAATCTATGGAAATCGACTACGAAGCTGCTATGGTTAAGAGA

406 **A A I H A A I K E E L D E K A**
1216 GCTGCTATCCACGCTGCTATCAAGGAAGAATTGGACGAAAAGGCT

421 **A K K E R L A G L S V A S V Q**
1261 GCTAAGAAGGAAAGATTGGCTGGTTTGTCTGTTGCTTCTGTTCAA

436 **N G T S G R Y R P V L M A V A**
1306 AACGGTACTTCTGGTAGATACAGACCAGTTTTGATGGCTGTTGCT

451 **T S G G G L I N Q H F G H A T**
1351 ACTTCTGGTGGTGGTTTGATCAACCAACACTTCGGTCACGCTACT

466 **E F L V Y E A S P S G V R F I**
1396 GAATTCTTGGTTTACGAAGCTTCTCCATCTGGTGTTAGATTCATC

481 **G H R R V D Q Y C V G N D T C**
1441 GGTCACAGAAGAGTTGACCAATACTGTGTTGGTAACGACACTTGT

496 **G E K E S A L A G S I R A L K**
1486 GGTGAAAAGGAATCTGCTTTGGCTGGTTCTATCAGAGCTTTGAAG

511 **G C E A V L C S K I G F E P W**
1531 GGTTGTGAAGCTGTTTTGTGTTCTAAGATCGGTTTCGAACCATGG

526 **S D L E T A G I Q P N G E H A**
1576 TCTGACTTGGAAACTGCTGGTATCCAACCAAACGGTGAACACGCT

541 **M E P I E E A V M A V Y R E M**
1621 ATGGAACCAATCGAAGAAGCTGTTATGGCTGTTTACAGAGAAATG

556 **I E S G R L E N D G A L L Q A**
1666 ATCGAATCTGGTAGATTGGAAAACGACGGTGCTTTGTTGCAAGCT

571 **K A** L E D Q F D P M V S K G E
1711 AAGGCGCTAGAGGATCAATTCGATCCCATGGTGAGCAAGGGCGAG

586 E L F T G V V P I L V E L D G
1756 GAGCTGTTCACCGGGGTGGTGCCCATCCTGGTCGAGCTGGACGGC

601 D V N G H K F S V S G E G E G
1801 GACGTAAACGGCCACAAGTTCAGCGTGTCCGGCGAGGGCGAGGGC

616 D A T Y G K L T L K F I C T T
1846 GATGCCACCTACGGCAAGCTGACCCTGAAGTTCATCTGCACCACC

631 G K L P V P W P T L V T T F T
1891 GGCAAGCTGCCCGTGCCCTGGCCCACCCTCGTGACCACCTTCACC

646 Y G V Q C F S R Y P D H M K Q
1936 TACGGCGTGCAGTGCTTCAGCCGCTACCCCGACCACATGAAGCAG

661 H D F F K S A M P E G Y V Q E
1981 CACGACTTCTTCAAGTCCGCCATGCCCGAAGGCTACGTCCAGGAG

676 R T I F F K D D G N Y K T R A
2026 CGCACCATCTTCTTCAAGGACGACGGCAACTACAAGACCCGCGCC

691 E V K F E G D T L V N R I E L
2071 GAGGTGAAGTTCGAGGGCGACACCCTGGTGAACCGCATCGAGCTG

706 K G I D F K E D G N I L G H K
2116 AAGGGCATCGACTTCAAGGAGGACGGCAACATCCTGGGGCACAAG

721 L E Y N Y N S H N V Y I M A D
2161 CTGGAGTACAACTACAACAGCCACAACGTCTATATCATGGCCGAC

736 K Q K N G I K V N F K I R H N
2206 AAGCAGAAGAACGGCATCAAGGTGAACTTCAAGATCCGCCACAAC

751 I E D G S V Q L A D H Y Q Q N
2251 ATCGAGGACGGCAGCGTGCAGCTCGCCGACCACTACCAGCAGAAC

766 T P I G D G P V L L P D N H Y
2296 ACCCCCATCGGCGACGGCCCCGTGCTGCTGCCCGACAACCACTAC

781 L S T Q S A L S K D P N E K R
2341 CTGAGCACCCAGTCCGCCCTGAGCAAAGACCCCAACGAGAAGCGC

796 D H M V L L E F V T A A G I T
2386 GATCACATGGTCCTGCTGGAGTTCGTGACCGCCGCCGGGATCACT

811 H G M D E L Y K *
2431 CACGGCATGGACGAGCTGTACAAGTAA

SU9-**NifB$_{Mi}$**-GFP

1 M A S T R V L A S R L A S Q M
1 ATGGCCTCCACTCGTGTCCTCGCCTCTCGCCTGGCCTCCCAGATG

16 A A S A K V A R P A V R V A Q
46 GCTGCTTCCGCCAAGGTTGCCCGCCCTGCTGTCCGCGTTGCTCAG

31 V S K R T I Q T G S P L Q T L
91 GTCAGCAAGCGCACCATCCAGACTGGCTCCCCCCTCCAGACCCTC

46 K R T Q M T S I V N A T T R Q
136 AAGCGCACCCAGATGACCTCCATCGTCAACGCCACCACCCGCCAG

61 A F Q K R A Y S S **M E K M S K**
181 GCTTTCCAGAAGCGCGCCTACTCTTCCATGGAGAAAATGTCTAAA

```
 76 F  S  H  L  L  K  A  H  P  C  F  N  E  K  V
226 TTTTCTCATTTGCTTAAGGCTCACCCATGTTTCAACGAAAAGGTT

 91 H  D  K  Y  G  R  V  H  L  P  V  A  P  R  C
271 CATGATAAGTATGGTAGAGTTCACTTGCCAGTGGCTCCTAGGTGC

106 N  I  A  C  K  F  C  K  R  S  V  S  K  E  C
316 AACATCGCATGTAAATTTTGCAAGAGATCTGTTTCAAAGGAATGT

121 C  E  H  R  P  G  V  S  L  G  V  L  K  P  E
361 TGCGAGCATAGGCCAGGTGTTAGTTTGGGAGTGTTAAAGCCTGAA

136 D  V  E  D  Y  L  K  K  I  L  K  E  M  P  N
406 GATGTGGAGGACTATTTGAAGAAAATTCTTAAGGAAATGCCAAAT

151 I  K  V  V  G  I  A  G  P  G  D  S  L  F  N
451 ATTAAGGTTGTGGGTATCGCTGGTCCTGGAGATTCCTTGTTTAAT

166 K  E  T  F  E  T  L  K  I  I  D  E  K  F  P
496 AAGGAGACTTTCGAGACACTTAAAATTATCGACGAAAAGTTCCCA

181 N  L  I  K  C  I  S  T  N  G  L  L  L  S  K
541 AATCTTATTAAGTGTATCTCTACAAACGGACTTTTGCTTTCAAAA

196 Y  Y  K  D  L  A  N  L  N  V  R  T  I  T  V
586 TATTACAAGGATCTTGCTAATTTGAACGTTAGAACTATCACAGTT

211 T  V  N  A  I  K  P  E  I  L  E  K  I  V  D
631 ACCGTGAATGCAATTAAACCTGAAATCTTGGAGAAGATAGTTGAT

226 W  V  Y  Y  D  K  K  L  Y  R  G  L  E  G  A
676 TGGGTGTACTACGACAAAAAGTTGTACAGAGGTCTTGAAGGAGCT

241 K  L  L  I  E  K  Q  I  E  G  I  K  K  A  S
721 AAGCTTTTGATCGAAAAGCAAATCGAGGGAATTAAAAAGGCTAGT

256 E  E  D  F  I  I  K  I  N  T  V  L  I  P  E
766 GAAGAGGATTTCATAATTAAAATCAATACCGTTCTTATCCCTGAG

271 I  N  M  D  H  V  V  E  I  A  K  F  F  K  D
811 ATAAACATGGATCACGTTGTGGAAATCGCTAAGTTTTTCAAGGAC

286 Y  A  Y  V  Q  N  I  I  P  L  I  P  Q  Y  K
856 TACGCATACGTTCAAAACATCATACCATTGATCCCTCAGTATAAG
```

301 **M K E L R A P T C E E I K K V**
901 ATGAAAGAACTTAGAGCTCCAACTTGTGAAGAAATTAAGAAAGTT

316 **R K E C E K Y I P Q F R A C G**
946 AGAAAGGAATGCGAGAAGTACATTCCTCAATTCAGAGCATGTGGA

331 **Q C R A D A V G L I K E K E L**
991 CAGTGCAGGGCTGATGCAGTGGGATTGATTAAAGAAAAGGAGCTT

346 **L K E F F K E K N K E K N I K**
1036 CTTAAGGAATTTTTCAAGGAAAAGAATAAGGAAAAGAACATCAAA

361 **L E V F D L K H F S H** A L E D
1081 CTTGAAGTTTTCGACTTGAAGCATTTCTCACACGCGCTAGAGGAT

376 Q F D P M V S K G E E L F T G
1126 CAATTCGATCCCATGGTGAGCAAGGGCGAGGAGCTGTTCACCGGG

391 V V P I L V E L D G D V N G H
1171 GTGGTGCCCATCCTGGTCGAGCTGGACGGCGACGTAAACGGCCAC

406 K F S V S G E G E G D A T Y G
1216 AAGTTCAGCGTGTCCGGCGAGGGCGAGGGCGATGCCACCTACGGC

421 K L T L K F I C T T G K L P V
1261 AAGCTGACCCTGAAGTTCATCTGCACCACCGGCAAGCTGCCCGTG

436 P W P T L V T T F T Y G V Q C
1306 CCCTGGCCCACCCTCGTGACCACCTTCACCTACGGCGTGCAGTGC

451 F S R Y P D H M K Q H D F F K
1351 TTCAGCCGCTACCCCGACCACATGAAGCAGCACGACTTCTTCAAG

466 S A M P E G Y V Q E R T I F F
1396 TCCGCCATGCCCGAAGGCTACGTCCAGGAGCGCACCATCTTCTTC

481 K D D G N Y K T R A E V K F E
1441 AAGGACGACGGCAACTACAAGACCCGCGCCGAGGTGAAGTTCGAG

496 G D T L V N R I E L K G I D F
1486 GGCGACACCCTGGTGAACCGCATCGAGCTGAAGGGCATCGACTTC

511 K E D G N I L G H K L E Y N Y
1531 AAGGAGGACGGCAACATCCTGGGGCACAAGCTGGAGTACAACTAC

526 N S H N V Y I M A D K Q K N G
1576 AACAGCCACAACGTCTATATCATGGCCGACAAGCAGAAGAACGGC

541 I K V N F K I R H N I E D G S

1621 ATCAAGGTGAACTTCAAGATCCGCCACAACATCGAGGACGGCAGC

556 V Q L A D H Y Q Q N T P I G D

1666 GTGCAGCTCGCCGACCACTACCAGCAGAACACCCCCATCGGCGAC

571 G P V L L P D N H Y L S T Q S

1711 GGCCCCGTGCTGCTGCCCGACAACCACTACCTGAGCACCCAGTCC

586 A L S K D P N E K R D H M V L

1756 GCCCTGAGCAAAGACCCCAACGAGAAGCGCGATCACATGGTCCTG

601 L E F V T A A G I T H G M D E

1801 CTGGAGTTCGTGACCGCCGCCGGGATCACTCACGGCATGGACGAG

616 L Y K *

1846 CTGTACAAGTAA

COX4-**twinStrep**-GFP

1 M L S L R Q S I R F F K P A T

1 ATGCTTTCACTTAGACAATCTATTAGATTTTTCAAGCCAGCTACA

16 R T L C S S R Y L L Q Q K P S

46 AGAACTTTGTGTTCTTCTAGATATCTTCTTCAGCAAAAACCTTCA

31 A **W S H P Q F E K G G G S G G**

91 GCATGGAGTCATCCTCAGTTTGAGAAAGGTGGAGGTTCAGGTGGT

46 **G S G G S A W S H P Q F E K** G

136 GGAAGCGGTGGATCTGCTTGGTCACATCCACAATTTGAAAAAGGA

61 S M V S K G E E L F T G V V P

181 TCCATGGTGAGCAAGGGCGAGGAGCTGTTCACCGGGGTGGTGCCC

76 I L V E L D G D V N G H K F S

226 ATCCTGGTCGAGCTGGACGGCGACGTAAACGGCCACAAGTTCAGC

91 V S G E G E G D A T Y G K L T

271 GTGTCCGGCGAGGGCGAGGGCGATGCCACCTACGGCAAGCTGACC

106 L K F I C T T G K L P V P W P

316 CTGAAGTTCATCTGCACCACCGGCAAGCTGCCCGTGCCCTGGCCC

121 T L V T T F T Y G V Q C F S R

361 ACCCTCGTGACCACCTTCACCTACGGCGTGCAGTGCTTCAGCCGC

136 Y P D H M K Q H D F F K S A M
406 TACCCCGACCACATGAAGCAGCACGACTTCTTCAAGTCCGCCATG

151 P E G Y V Q E R T I F F K D D
451 CCCGAAGGCTACGTCCAGGAGCGCACCATCTTCTTCAAGGACGAC

166 G N Y K T R A E V K F E G D T
496 GGCAACTACAAGACCCGCGCCGAGGTGAAGTTCGAGGGCGACACC

181 L V N R I E L K G I D F K E D
541 CTGGTGAACCGCATCGAGCTGAAGGGCATCGACTTCAAGGAGGAC

196 G N I L G H K L E Y N Y N S H
586 GGCAACATCCTGGGGCACAAGCTGGAGTACAACTACAACAGCCAC

211 N V Y I M A D K Q K N G I K V
631 AACGTCTATATCATGGCCGACAAGCAGAAGAACGGCATCAAGGTG

226 N F K I R H N I E D G S V Q L
676 AACTTCAAGATCCGCCACAACATCGAGGACGGCAGCGTGCAGCTC

241 A D H Y Q Q N T P I G D G P V
721 GCCGACCACTACCAGCAGAACACCCCCATCGGCGACGGCCCCGTG

256 L L P D N H Y L S T Q S A L S
766 CTGCTGCCCGACAACCACTACCTGAGCACCCAGTCCGCCCTGAGC

271 K D P N E K R D H M V L L E F
811 AAAGACCCCAACGAGAAGCGCGATCACATGGTCCTGCTGGAGTTC

286 V T A A G I T H G M D E L Y K
856 GTGACCGCCGCCGGGATCACTCACGGCATGGACGAGCTGTACAAG

301 *
901 TAA
901
901 ATT

COX4-**twinStrep**-$NifB_{Av}$

1 M L S L R Q S I R F F K P A T
1 ATGCTTTCACTTAGACAATCTATTAGATTTTTCAAGCCAGCTACA

16 R T L C S S R Y L L Q Q K P S
46 AGAACTTTGTGTTCTTCTAGATATCTTCTTCAGCAAAAACCTTCA

31 A **W S H P Q F E K G G G S G G**
91 GCATGGAGTCATCCTCAGTTTGAGAAAGGTGGAGGTTCAGGTGGT

46 **G S G G S A W S H P Q F E K** G
136 GGAAGCGGTGGATCTGCTTGGTCACATCCACAATTTGAAAAAGGA

61 S M E L S V L G Q N N G G Q H
181 TCCATGGAATTGTCTGTCTTGGGTCAAAACAATGGTGGTCAACAT

76 S A G G C S S S S C G S T H D
226 TCTGCTGGTGGTTGTTCTTCTTCATCTTGTGGTTCTACTCACGAT

91 Q L S H L P E N I R A K V Q N
271 CAATTGTCTCATTTGCCAGAAAACATTAGAGCCAAGGTTCAAAAC

106 H P C Y S E E A H H Y F A R M
316 CATCCATGCTATTCTGAAGAAGCCCATCATTACTTTGCTAGAATG

121 H V A V A P A C N I Q C H Y C
361 CATGTTGCTGTTGCTCCAGCTTGTAACATTCAATGTCATTACTGC

136 N R K Y D C A N E S R P G V V
406 AACAGAAAGTACGATTGCGCTAATGAATCTAGACCAGGTGTTGTT

151 S E V L T P E Q A V K K V K A
451 TCTGAAGTCTTGACTCCAGAACAAGCTGTTAAGAAAGTTAAGGCT

166 V A A A I P Q M S V L G I A G
496 GTTGCTGCTGCTATTCCACAAATGTCTGTTTTAGGTATTGCTGGT

181 P G D P L A N P K R T L D T F
541 CCAGGTGATCCATTGGCTAATCCAAAAAGAACTTTGGACACTTTC

196 R M L S E Q A P D I K L C V S
586 AGGATGTTGTCTGAACAAGCTCCAGATATTAAGTTGTGCGTTTCT

211 T N G L A L P E C V E E L A K
631 ACTAATGGTTTGGCCTTGCCAGAATGTGTTGAAGAATTGGCTAAG

226 H N I D H V T I T I N C V D P
676 CACAACATCGATCATGTTACCATTACCATCAACTGCGTTGATCCA

241 E I G A K I Y P W I Y W N N K
721 GAAATTGGTGCTAAAATCTACCCATGGATCTACTGGAACAACAAG

256 R I R G V K A A K I L I E Q Q
766 AGAATTAGAGGTGTTAAGGCCGCCAAGATTTTGATCGAACAACAA

271 Q K G L E M L V A R G I L V K
811 CAAAAAGGCTTGGAAATGTTGGTTGCCAGAGGTATTTTGGTTAAG

286 V N S V M I P G V N D E H L K

856 GTTAACTCCGTTATGATCCCAGGTGTTAACGATGAACACTTGAAA

301 E V S K I V K A K G A F L H N

901 GAAGTTTCCAAGATCGTTAAGGCTAAGGGTGCTTTCTTGCATAAT

316 V M P L I A E P E H G T F Y G

946 GTTATGCCATTGATTGCCGAACCAGAACATGGTACTTTTTATGGT

331 V M G Q R S P E P E E L Q D L

991 GTTATGGGTCAGAGATCTCCAGAACCAGAAGAATTGCAAGACTTA

346 Q D A C A G D M N M M R H C R

1036 CAAGATGCTTGTGCTGGTGATATGAATATGATGAGACATTGCAGA

361 Q C R A D A V G M L G E D R G

1081 CAATGCAGAGCTGATGCTGTTGGTATGTTGGGTGAAGATAGAGGT

376 D E F T L D K I E S M E I D Y

1126 GATGAATTCACCTTGGATAAGATCGAGTCCATGGAAATTGATTAC

391 E A A M V K R A A I H A A I K

1171 GAAGCTGCTATGGTTAAGAGAGCTGCAATTCACGCTGCTATCAAA

406 E E L D E K A A K K E R L A G

1216 GAAGAATTAGACGAAAAGGCTGCCAAGAAAGAAAGATTGGCTGGT

421 L S V A S V Q N G T S G R Y R

1261 TTGTCTGTTGCCTCTGTTCAAAATGGTACTTCTGGTAGATATAGG

436 P V L M A V A T S G G G L I N

1306 CCAGTTTTGATGGCTGTTGCAACTTCAGGTGGTGGTTTGATCAAT

451 Q H F G H A T E F L V Y E A S

1351 CAACATTTTGGTCATGCTACCGAGTTCTTGGTTTATGAAGCTTCT

466 P S G V R F I G H R R V D Q Y

1396 CCATCTGGTGTTAGATTCATCGGTCATAGAAGAGTTGATCAATAC

481 C V G N D T C G E K E S A L A

1441 TGCGTTGGTAACGATACCTGTGGTGAAAAAGAATCTGCTTTGGCA

496 G S I R A L K G C E A V L C S

1486 GGTTCTATTAGAGCTTTGAAAGGTTGTGAAGCTGTCTTGTGTTCT

511 K I G F E P W S D L E T A G I

1531 AAGATTGGTTTCGAACCATGGTCTGATTTGGAAACTGCTGGTATT

526 Q P N G E H A M E P I E E A V
1576 CAACCTAACGGTGAACATGCTATGGAACCTATTGAAGAGGCTGTT

541 M A V Y R E M I E S G R L E N
1621 ATGGCTGTTTACAGAGAAATGATTGAATCCGGTAGATTGGAAAAC

556 D G A L L Q A K A *
1666 GATGGTGCATTATTGCAAGCTAAGGCTTGA

COX4-**twinStrep**-NifB$_{Mi}$

1 M L S L R Q S I R F F K P A T
1 ATGCTTTCACTTAGACAATCTATTAGATTTTTCAAGCCAGCTACA

16 R T L C S S R Y L L Q Q K P S
46 AGAACTTTGTGTTCTTCTAGATATCTTCTTCAGCAAAAACCTTCA

31 A **W S H P Q F E K G G G S G G**
91 GCATGGAGTCATCCTCAGTTTGAGAAAGGTGGAGGTTCAGGTGGT

46 **G S G G S A W S H P Q F E K** G
136 GGAAGCGGTGGATCTGCTTGGTCACATCCACAATTTGAAAAAGGA

61 S M E K M S K F S H L L K A H
181 TCCATGGAAAAGATGTCCAAGTTCTCCCATTTGTTGAAAGCTCAT

76 P C F N E K V H D K Y G R V H
226 CCATGCTTCAACGAAAAGGTTCATGATAAGTACGGTAGAGTTCAT

91 L P V A P R C N I A C K F C K
271 TTGCCAGTTGCTCCAAGATGTAACATTGCTTGTAAGTTCTGCAAG

106 R S V S K E C C E H R P G V S
316 AGGTCCGTTTCTAAAGAATGTTGTGAACATAGACCAGGTGTTTCT

121 L G V L K P E D V E D Y L K K
361 TTGGGTGTTTTGAAACCAGAAGATGTTGAGGACTACCTGAAAAAG

136 I L K E M P N I K V V G I A G
406 ATCTTGAAAGAGATGCCAAACATCAAGGTTGTTGGTATTGCTGGT

151 P G D S L F N K E T F E T L K
451 CCTGGTGATTCTCTGTTTAACAAAGAAACTTTCGAAACCCTGAAG

166 I I D E K F P N L I K C I S T
496 ATCATCGACGAAAAGTTTCCCAACTTGATTAAGTGCATTTCCACC

```
 181 N  G  L  L  L  S  K  Y  Y  K  D  L  A  N  L
 541 AACGGTCTGTTGTTGTCTAAGTACTACAAGGATTTGGCCAACTTG

 196 N  V  R  T  I  T  V  T  V  N  A  I  K  P  E
 586 AACGTTAGAACTATTACCGTTACTGTCAACGCCATTAAGCCAGAA

 211 I  L  E  K  I  V  D  W  V  Y  Y  D  K  K  L
 631 ATCTTGGAAAAAATCGTTGACTGGGTTTACTACGACAAGAAGTTG

 226 Y  R  G  L  E  G  A  K  L  L  I  E  K  Q  I
 676 TATAGAGGTTTGGAAGGTGCCAAGTTGTTGATCGAAAAACAAATC

 241 E  G  I  K  K  A  S  E  E  D  F  I  I  K  I
 721 GAAGGTATCAAGAAGGCCTCCGAAGAAGATTTCATTATCAAGATC

 256 N  T  V  L  I  P  E  I  N  M  D  H  V  V  E
 766 AACACCGTCTTGATCCCAGAAATCAACATGGATCACGTTGTTGAA

 271 I  A  K  F  F  K  D  Y  A  Y  V  Q  N  I  I
 811 ATTGCCAAGTTCTTCAAGGATTACGCCTACGTTCAAAACATCATT

 286 P  L  I  P  Q  Y  K  M  K  E  L  R  A  P  T
 856 CCATTGATTCCACAGTACAAGATGAAGGAATTGAGAGCACCAACT

 301 C  E  E  I  K  K  V  R  K  E  C  E  K  Y  I
 901 TGCGAAGAAATCAAAAAGGTCAGAAAAGAGTGCGAGAAGTACATC

 316 P  Q  F  R  A  C  G  Q  C  R  A  D  A  V  G
 946 CCACAATTCAGAGCTTGTGGTCAATGTAGAGCTGATGCTGTTGGT

 331 L  I  K  E  K  E  L  L  K  E  F  F  K  E  K
 991 CTGATCAAAGAAAAAGAGCTGTTGAAAGAGTTTTTCAAAGAGAAG

 346 N  K  E  K  N  I  K  L  E  V  F  D  L  K  H
1036 AACAAAGAAAAGAACATCAAGCTGGAAGTGTTCGACTTGAAGCAC

 361 F  S  H  *
1081 TTCTCTCATTGA
```

SUPPLEMENTARY FIGURE 8. DNA and protein sequences of SU9 and COX4 mitochondria targeted NifB variants. DNA and translated protein sequences of NifB variants targeted to the mitochondria of *N. benthamiana* using the SU9 or COX4 leader sequences from plant expression vectors shown in Supplementary Figure 7 and Supplementary Figure 10.

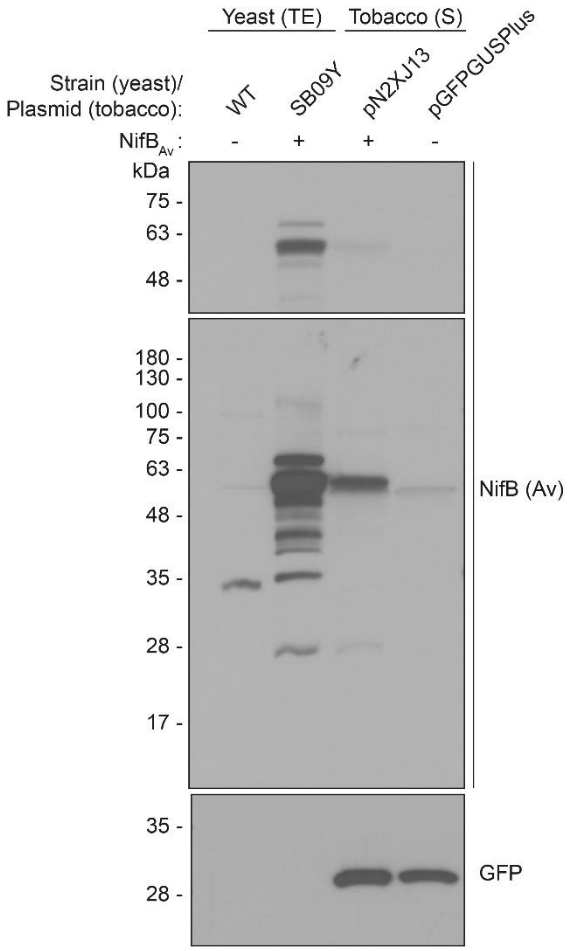

SUPPLEMENTARY FIGURE 9. Expression and solubility of mitochondria targeted (SU9) NifB$_{Av}$ in *N. benthamiana* leaves. Full-size image of cropped exposure in Figure 6B.

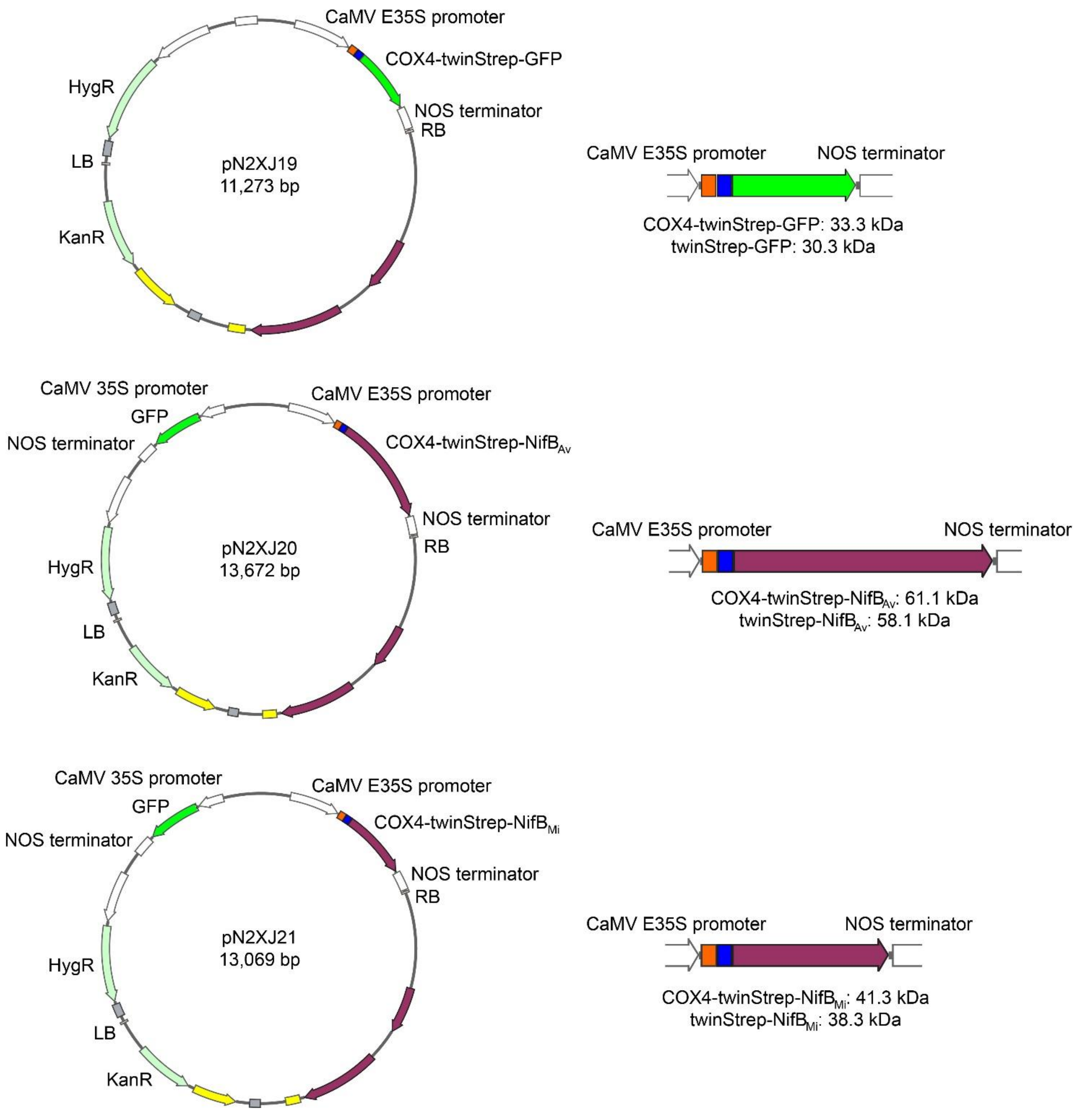

SUPPLEMENTARY FIGURE 10. Plant expression vectors with COX4 leader sequence. Schematic overview of plant expression vectors for expression of COX4-twinStrep-GFP, COX4-twinStrep-NifB$_{Av}$ and COX4-twinStrep-NifB$_{Mi}$. See Supplementary Figure 8 for detailed information about DNA and protein sequences.
